# A Nutrient-Dependent ANGPTL3–PCSK9-Containing Complex in HepG2 Cells and Human Plasma

**DOI:** 10.3390/biom16101531

**Published:** 2026-10-20

**Authors:** Simone Bini, Valeria Pecce, Alessandra Pinzon Grimaldos, Alessia Di Costanzo, Ilenia Minicocci, Stella Covino, Daniele Tramontano, Silvia Piconese, Laura D’Erasmo, Marcello Arca

**Affiliations:** 1 Department of Translational and Precision Medicine, Sapienza University of Rome, 00161 Rome, Italy; simone.bini@uniroma1.it (S.B.);; 2 Unità di Neuroimmunologia, IRCCS Fondazione Santa Lucia, 00175 Rome, Italy

**Keywords:** ANGPTL3, PCSK9, nutrients, ANGPTL3-PCSK9 complex, lipid metabolism

## Abstract

ANGPTL3 and PCSK9 are key regulators of lipoprotein metabolism, with emerging evidence suggestive of a functional regulatory interrelationship. Indeed, patients lacking ANGPTL3 due to loss-of-function variants exhibit reduced circulating PCSK9 levels. This study aimed to determine whether ANGPTL3 and PCSK9 are part of the same complex. Co-immunoprecipitation (Co-IP) experiments in HepG2 cells and human plasma assessed whether ANGPTL3 and PCSK9 co-immunoprecipitated. A hypothetical interaction between the two proteins was also modeled in silico using two software packages: AlphaFold2 and ZDOCK. HepG2 cells overexpressing ANGPTL3, PCSK9, and ANGPTL3-PCSK9 were generated to investigate the relationship between ANGPTL3 and PCSK9 under different nutrient conditions. Reciprocal Co-IP experiments in HepG2 cells and human plasma showed that ANGPTL3 and PCSK9 could be recovered in the same co-immunoprecipitated sample, both intracellularly and in the culture medium. Structural modeling supported the experimental observations and identified a 2:1 ANGPTL3:PCSK9 arrangement as a plausible configuration. We observed higher association under fasting conditions and lower association under simulated feeding conditions. The latter was also confirmed in postprandial human plasma, where ANGPTL3 is known to bind ANGPTL8 in a 2:1 complex. Moreover, incubation of HepG2 cells with LDL appeared to reduce ANGPTL3-PCSK9 association. In summary, our study identifies a dynamic ANGPTL3–PCSK9 association that is regulated in a nutrient-dependent fashion. These findings may broaden current understanding of ANGPTL3 and PCSK9 biology and support further mechanistic studies, particularly in the context of therapeutic inhibition of these proteins.

## 1. Introduction

Angiopoietin-like protein 3 (ANGPTL3) and proprotein convertase subtilisin/kexin type 9 (PCSK9) are key regulators of plasma lipoprotein metabolism [1,2,3]. Both proteins are predominantly produced by the liver and contribute to the regulation of circulating lipoproteins through distinct but potentially interconnected pathways. In the extracellular space, ANGPTL3 inhibits lipases, particularly lipoprotein lipase and endothelial lipase [4]. Through these actions, ANGPTL3 plays a pivotal role in the removal of triglyceride-rich lipoproteins (TRLs), very-low-density lipoproteins (VLDL), and chylomicrons, as well as in the catabolism of high-density lipoproteins (HDL) [3,5]. In particular, by regulating TRL lipolysis during fasting and postprandial states, ANGPTL3 also contributes to the distribution of lipid energy substrates between storage tissues and highly metabolically active tissues [3]. 

The metabolic activity of ANGPTL3 is closely linked to nutritional status through its interaction with other members of the ANGPTL family, particularly ANGPTL8. During fasting, ANGPTL8 expression is low and ANGPTL3 has relatively limited LPL-inhibitory activity. After feeding, hepatic ANGPTL8 expression increases and ANGPTL8 associates with ANGPTL3, markedly enhancing the inhibition of LPL in oxidative tissues and favoring the redistribution of circulating triglyceride-derived fatty acids toward adipose tissue for storage [3,5]. Thus, ANGPTL3 participates in a nutrient-responsive network that coordinates lipid utilization and storage during the fasting–feeding transition. This concept has led to the description of the ANGPTL3–4–8 axis as an important physiological regulator of tissue-specific lipid partitioning [3,5].

These effects in humans have been firmly established by studies of individuals harboring loss-of-function (LOF) variants in the ANGPTL3 gene. These individuals show markedly reduced plasma concentrations of TRLs, HDL, and low-density lipoprotein cholesterol (LDL-C), a phenotype known as familial combined hypobetalipoproteinemia type 2 (FHBL2) [6]. FHBL2 is an ultra-rare condition, and affected individuals appear to be protected from cardiovascular disease [6,7,8]. These observations, together with the lipid-lowering effects of pharmacological ANGPTL3 inhibition, indicate that ANGPTL3 influences several pathways of lipoprotein metabolism beyond its canonical regulation of LPL activity.

PCSK9 belongs to the superfamily of kexin-like proteases, but its proteolytic activity is lost during protein maturation. Indeed, the PCSK9 propeptide is autocatalytically cleaved in the endoplasmic reticulum, folds separately from the rest of the protein, and remains bound to its catalytic site, thereby inactivating the protein [9,10]. The main function of PCSK9 is to regulate plasma LDL-C levels by modulating the recycling of the low-density lipoprotein receptor (LDLR). PCSK9 can form a complex with LDLR, probably extracellularly, thereby regulating LDLR turnover and recycling to the cell membrane [11,12]. 

Importantly, PCSK9 is also metabolically regulated. Hepatic PCSK9 expression is controlled by sterol-sensitive transcriptional pathways, particularly SREBP2 and HNF1α, thereby linking PCSK9 production to intracellular cholesterol homeostasis. Experimental studies have also suggested a contribution of insulin- and SREBP1c-dependent pathways to the nutritional regulation of PCSK9 expression [13]. In humans, circulating PCSK9 displays a diurnal rhythm and decreases substantially during prolonged fasting, in parallel with reduced endogenous cholesterol synthesis [14,15]. Conversely, the response to refeeding and acute nutritional stimuli appears to depend on the composition and duration of the dietary challenge. These observations indicate that PCSK9, like the ANGPTL3–4–8 system, participates in the metabolic adaptation to changes in nutrient availability.

The functions summarized above suggest that these two proteins may act in distinct cellular compartments, influencing different lipid and lipoprotein pathways. ANGPTL3 has traditionally been associated mainly with regulation of extracellular lipases and triglyceride partitioning, whereas PCSK9 primarily regulates LDLR abundance and LDL clearance. Nevertheless, increasing evidence suggests that these pathways may not be completely independent. Surprisingly, Fazio et al. [16] found reduced levels of circulating PCSK9 in patients carrying homozygous LOF in ANGPTL3, thus suggesting a possible mutual regulation between ANGPTL3 and PCSK9. Additionally, studies in individuals with ANGPTL3 activity inhibited by monoclonal antibodies suggested that this protein may also contribute to the LDLR-dependent clearance of ApoB-containing lipoproteins [17].

Taken together, these observations raise the possibility that ANGPTL3 and PCSK9 participate in a previously unrecognized regulatory network linking triglyceride partitioning, LDLR activity, and nutritional status. However, the possibility that ANGPTL3 and PCSK9 may be part of the same complex has not been investigated. In particular, whether their association changes during the transition between fasting and feeding remains unknown. Based on the evidence outlined above, this study was designed to investigate whether ANGPTL3 and PCSK9 may be found in a complex and how their association or regulation is influenced by nutrient conditions.

## 2. Materials and Methods

### 2.1. Cellular Models

HepG2 cell lines (provided by ATCC #HB-8065) were cultured in Dulbecco’s Modified Eagle Medium (Gibco-BRL Division, Thermo Fisher Scientific, Waltham, MA, USA, #11965092) containing 10% fetal bovine serum (Gibco-BRL Division, Thermo Fisher Scientific, Waltham, MA, USA, #10100147) and antibiotic–antimycotic solution (Gibco-BRL Division, Thermo Fisher Scientific, Waltham, MA, USA, #15140122) and incubated at 37 °C in an atmosphere of 5% CO_2_. We generated the *ANGPTL3* and *PCSK9* overexpressing lines using plasmids containing the complementary DNA (cDNA) of *ANGPTL3* (NM_014495) or *PCSK9* (NM_174936) under the control of the constitutive cytomegalovirus (CMV) promoter. OriGene provided both pCMV-ANGPTL3 and pCMV-PCKS9 plasmids. Cell lines were transfected with empty vector or pCMV-ANGPTL3, pCMV-PCSK9, or both plasmids using Lipofectamine 3000 (Invitrogen Division, Thermo Fisher Scientific, Waltham, MA, USA, #L3000015) according to the manufacturer’s instructions. Briefly, cells were plated the day before transfection at 70% confluence in 6-well plates and starved for two hours before transfection. Cells were FBS-starved either in Dulbecco’s Modified Eagle Medium (Gibco-BRL Division, Thermo Fisher Scientific, Waltham, MA, USA, #11965092) to simulate a nutrient-rich condition, or Ham’s F12 (Gibco-BRL Division, Thermo Fisher Scientific, Waltham, MA, USA, #11320033) to simulate a nutrient-poor condition. The switch between nutrient-poor and nutrient-rich conditions was used to simulate feeding; conversely, switching from nutrient-rich to nutrient-poor medium was used to simulate fasting. Cells were harvested 48 h after the transfections.

### 2.2. Human Samples Preparation and Analysis

Subjects enrolled in this study were healthy subjects (defined by no chronic drug intake) aged between 18 and 80, and willing to participate in the study. Subjects carrying pathogenic variants in *LDLR*, *APOB*, *PCSK9* or *ANGPTL3* were excluded together with subjects suffering from chronic diseases. Subjects were invited to our lipid clinic, and anthropometric parameters such as height, weight, and waist circumference were measured. Blood samples were collected under fasting conditions, defined as at least 10 h after the last meal. Subjects were then submitted to a test meal (muffin prepared with olive oil, eggs, ricotta cheese, nuts, cocoa, wheat flour, and skimmed milk), which consisted of 73 g fat, 52 g carbohydrate, 22 g protein, 145 mg cholesterol, and 980 Kcal. The blood draw in feeding conditions was performed 4.5h after the test meal. The 3 healthy subjects harbored a wild-type genotype for *ANGPTL3*, *PCSK9*, *APOB* and *LDLR*. They participated in a previous postprandial study and agreed to participate in this new study [6]. The Ethical Committee of the Sapienza University of Rome and the Policlinico Umberto I approved the study protocol, and all study participants provided their informed consent (Protocol 0061/2022). All procedures involving human participants were conducted in accordance with the ethical principles of the Declaration of Helsinki. Blood samples were centrifuged at 4000 rpm for 15 min in a benchtop centrifuge, after which plasma was separated and analyzed under fasting or feeding conditions. Blood chemistry was performed in feeding and fasting conditions using the ILab 300 plus Clinical Chemistry System as per the manufacturer’s protocol; data are reported in Supplementary Table S1.

### 2.3. Protein Extraction

Total proteins were extracted from cell pellets with a lysis buffer containing Tris-HCl (pH 7.4, 50 Mm, #15567027), NaCl (150 mM, #12352302), Triton (1% *v*/*v*, #12161900), ethylenediaminetetraacetic acid (EDTA, 20 mM, #E6511), phenylmethylsulfonyl fluoride (PMSF, 2 mM, #12352202), protease and phosphatase inhibitors (Pierce, Rockford, IL, USA, #A32963), leupeptin (2 ug/mL, #12352202), and glycerol (10% *v*/*v*, #50161901). All extractions were done on ice, and the products were quantified using the Bradford method. Proteins from cell culture media were concentrated using StrataClean resin (Agilent). In particular, 2 mL of culture medium was incubated overnight with 10 μL of StrataClean resin in low-speed wheel rotation at 4 °C. After overnight incubation, samples were centrifuged at 4000 RPM for 4 min, the supernatant was discharged, and the resin pellet containing proteins was suspended in 20 uL of loading buffer and incubated for 5 min at 95 °C. Then, the samples were centrifuged at 4000 RPM for 4 min, and the supernatants were loaded into a polyacrylamide gel.

### 2.4. Co-Immunoprecipitation

Co-immunoprecipitations were performed using Dynabeads G (Thermofisher Scientific, #10004D). A total of 50 µL of Dynabeads were neutralized with a wash in phosphate-buffered Saline (PBS, #10010023) solution and then bound with 1 µg of specific antibody diluted 1:100 in PBS. Beads were then washed twice and resuspended in 100 µg of total protein extract in 1 mL of PBS in overnight gentle rotation at 4 °C. Beads were then magnetically separated from total proteins, the antibody–bead complex was dissociated using a loading buffer, and beads were boiled in a heat bath at 95 °C and loaded in SDS-polyacrylamide gel for electrophoretic separation as described in the Western blotting section. Co-immunoprecipitations in plasma were performed using 100 µL of plasma diluted in 2 mL of PBS.

### 2.5. Western Blotting

A total of 30 μg of total protein extract, or 10 uL of proteins isolated from the culture medium as described in the Protein extraction paragraph, were separated using a gradient (4–20%) sodium dodecyl sulfate-polyacrylamide gel (SDS-PAGE) and transferred onto a polyvinylidene difluoride (PVDF) membrane. Alternatively, proteins were separated using a gradient Bis-Tris (4–12%) MOPS buffer. After 2 h of blocking using non-fat dry milk at 5%, proteins were detected with specific primary antibodies at a dilution of 1:2000: ANGPTL3 (GeneTex, #GTX17219), ANGPTL8 (GeneTex, #GTX32128), PCSK9 (Abcam, #ab181142), β-ACTIN (Sigma Aldrich, #A1978), and Albumin (Abcam, #ab10241), which were incubated overnight at 4 °C. After the incubation with horseradish peroxidase (HRP)-conjugated secondary antibodies, the bands were detected with chemiluminescence using Clarity Western ECL substrate (BIO-RAD) and a charge-coupled-device camera (Chemidoc, BIO-RAD). Alternatively, the bands were detected with a colorimetric precipitate using alkaline-phosphatase (AP)-conjugated secondary antibodies and 5-bromo-4-chloro-3-indolyl-phosphate/nitro blue tetrazolium substrates (Promega, #S3721). 

### 2.6. Immunofluorescence

Immunofluorescence was performed using fixed cells in 4% paraformaldehyde (PFA, #158127) for 10 min, and then the cells were washed twice in phosphate-buffered solution (PBS, #10010023). Cells were then permeabilized using PBS-Triton-X solution 0.01%, and blocking was performed using a solution of PBS-bovine serum albumin (BSA) 3% for 2 h. Primary antibodies were then incubated overnight at a dilution of 1:50. The day after, secondary antibodies were incubated for 45 min, cells were washed twice, and nuclei were stained with the 4′,6-diamidino-2-phenylindole (DAPI) at a dilution of 1:5000. The fluorescence was conserved with a drop of mounting solution (1:1 PBS and glycerol) on samples and acquired using the THUNDER imaging system inverted microscope (Leica), using the 63X oil-immersion objective (theoretical lateral resolution in the range of approximately 200–250 nm). Colocalization between ANGPTL3 and PCSK9 fluorescence signals was assessed using a Python-based image analysis pipeline implemented with the scikit-image package. Individual blue, green, and red fluorescence channels were exported from the raw microscopy files as TIFF images and processed separately. Background signal was reduced using rolling-ball background subtraction. Each channel was then converted to 8-bit using percentile-based intensity scaling and moderately enhanced by contrast-limited adaptive histogram equalization (CLAHE) and unsharp masking while preserving the black background. Red/green colocalization was evaluated pixel by pixel after independent Otsu thresholding of the red and green channels. Pixels were classified as colocalization-positive when both red and green fluorescence intensities were above their respective thresholds. Blue-positive pixels were excluded from the final red/green colocalization mask to avoid nuclear or blue-channel-associated regions. The analysis generated merged RGB images and binary masks highlighting colocalization-positive regions, which were used for qualitative comparison between experimental conditions.

### 2.7. LDL Separation and Treatment

Low-density lipoproteins (LDLs) were isolated from a pooled plasma of healthy donors using serial ultracentrifugation. In brief, 4 mL of plasma was adjusted to a density of 1.005 g/mm^3^ and ultracentrifuged at 195.000× *g* for 24 h. After ultracentrifugation, the supernatant containing VLDL + chylomicrons was discharged, and the solution was further adjusted to a density of 1.067 g/mm^3^ using sodium bromide. Solution was further ultracentrifuged at 195.000× *g* for 24h. A total of 2 mL supernatant containing LDLs was collected. The LDL solution was then desalted using ion exchange columns PD-10 (Cytiva, #17-0851-01). LDL-cholesterol concentration was measured using ILab 300 plus Clinical Chemistry System and then concentrated using SpeedVac^®^ (Eppendorf, Boston, MA, USA) at 10 µg/µL. In conclusion, the solution was sterile-filtered using 0.2 µm filter (Sigma) and then stored for cellular use. In this paper, cells were treated for 4h with human LDL at a final concentration of 10 µg/mL.

### 2.8. Flow Cytometry

Cells were labeled with the fixable viability dye eFluor780 (ThermoFisher Scientific, #65-0865). Then, they were stained for surface markers with LDLR BV605 (BD, #745174). All flow cytometry experiments were acquired on LSR Fortessa (BD Biosciences, San Josè, CA, USA). Data was analyzed using FlowJo software, version 10.0.8r1 (BD Biosciences). Data were selected as follows for the gating strategy: live cells were selected based on Viability Dye and FSC-A, and doublets were excluded using FSC-A versus FSC-W. Single HepG2 cells were selected and analyzed using FlowJo software (v.10.9.0). For each event, LDLR fluorescence was measured at 600 nm, and the mean geometric fluorescence per each condition was calculated and reported in Supplementary Figure S3.

### 2.9. In Silico Prediction of ANGPTL3-PCSK9 Complex Formation

We used AphaFold2 and ZDOCK software to verify the possible interaction between ANGPTL3 and PCSK9 and to model a potential molecular docking into a bigger complex. AlphaFold2 produces a per-residue confidence metric called the predicted local distance difference test (pLDDT) on a scale from 0 to 100 [18]. The pLDDT estimates how well the prediction would agree with an experimental structure based on the local distance difference test Cα (lDDT-Cα) [19]. The program computes the potential existence of the spatial distribution of the single residue sidechains in association with the potential position of the nearby residues based on existing crystallographic repositories in the Protein Data Bank (PDB) [18]. A pLDDT > 90 is considered very accurate, and a pLDDT ≥ 70 is considered accurate [18,19]. Deriving from single residues’ pLDDTs, the software computes the predicted template model score (pTM). This is a metric for the similarity between protein structures (in this case, between the predicted structure and the assumed real structure). The model is considered accurate if pTM > 0.50; for a pTM = 0.50 the P-value for the existence of the model is 5.5 × 10^−7^ [20]. Similarly to pTM, the interface predicted model score (ipTM) computes the potential existence of the single residue sidechain position in comparison to the interfacing one, considering the positions of the remaining sidechains, like the pTM. Also, a structure having ipTM > 0.50 is considered accurate [21]. ZDOCK software, independently from AlphaFold2, predicts the existence of a protein–protein interaction or multimer by using the deposited PDB data of the specific interacting proteins through the analysis of both spatial and electrostatic hindrance [22]. For sequence references, we used the corresponding UniProt entries. For ANGPTL3, we used UniProt entry Q9Y5C1. We removed the N-terminal signal peptide (amino acids 1–16), which is cleaved during protein maturation, and used the processed sequence 17–460 as input for both AlphaFold2 and ZDOCK (UniProt feature reference: PRO_0000009122). For PCSK9, we used UniProt entry Q8NBP7. We removed the N-terminal signal peptide (amino acids 1–30) and modeled PCSK9 in its physiologically processed conformation, consisting of the prodomain (31–152; PRO_0000027120) and mature PCSK9 (153–692; PRO_0000027121). We chose this representation because PCSK9 is known to undergo autocatalytic cleavage of its prodomain, which remains associated with the mature protein and occupies the catalytic site, thereby maintaining the protein in an enzymatically inactive conformation [23]. For ANGPTL8, we used UniProt entry Q6UXH0. We removed the N-terminal signal peptide (amino acids 1–21) and used the processed sequence 22–198 as model input (UniProt feature reference: PRO_0000319617). Full FASTA sequences used for structural modeling are now provided in Supplementary Data S1.

### 2.10. Quantitative PCR

RNA was isolated from cells using the RNeasy Mini Kit (Qiagen, Hilden, Germany, #74104) and quantified with the Nanodrop 2000 (Thermo Fisher Scientific). The High-Capacity cDNA Reverse Transcription Kit (Thermo Fisher Scientific,#4368814) was used to synthesize cDNA from RNA according to the manufacturer’s instructions (Thermo Fisher Scientific). The expression levels of *ANGPTL3* and *PCSK9* were analyzed in cDNAs from treated and control HepG2 cells using TaqMan Gene Expression assays and TaqMan Universal Master Mix according to the manufacturer’s instructions (Thermo Fisher Scientific). Results were calculated using the 2−ΔΔCt method and normalized using β-Actin as endogenous control (TaqMan^®^ Gene Expression Assays HS99999903). Data were expressed as the mean ± standard deviation (SD) of three replicates relative to the CTRL sample that was used as a calibrator.

### 2.11. Statistical Analysis

Statistical analyses were performed using GraphPad Prism (version 8.0.1). Differences between continuous variables were assessed using an unpaired two-tailed Student’s *t*-test for cell-line experiments. For experiments with multiple matched samples, data was analyzed using paired tests and expressed relative to the control condition. Two-way ANOVA was used to analyze flow cytometry data. A *p*-value < 0.05 was considered statistically significant.

## 3. Results

### 3.1. ANGPTL3 and PCSK9 Can Be Found in the Same Co-IP Samples Intracellularly and Extracellularly

**Figure 1:**
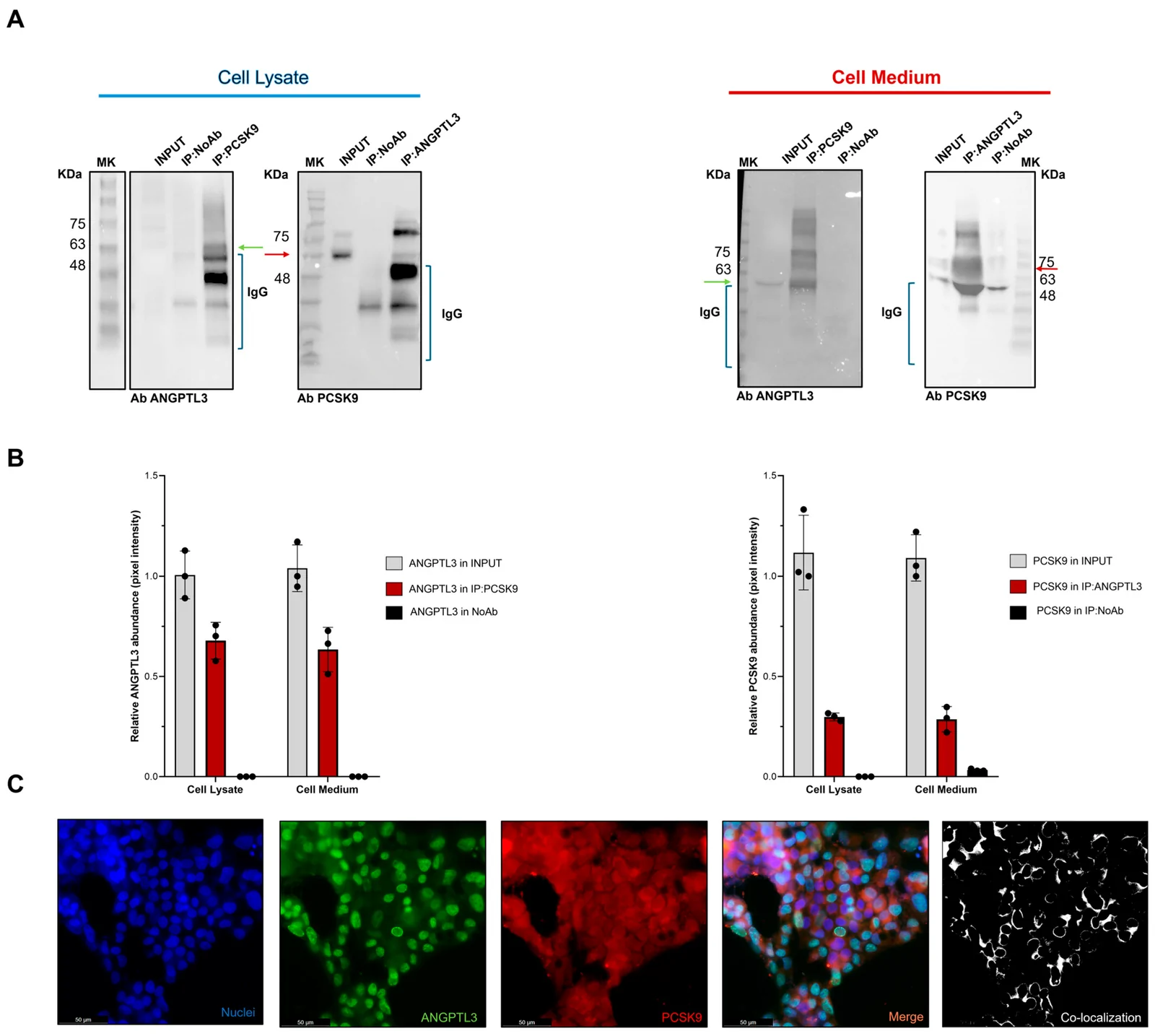
Reciprocal co-immunoprecipitation and co-localization of ANGPTL3 and PCSK9 in HepG2 cells. (**A**) shows representative Co-Immunoprecipitation (Co-IP) of total proteins isolated from cell lysate and cell medium. Cell lysate represents Co-IP of 100 µg of total protein extract from HepG2 cells. IP:ANGPTL3 and IP:PCSK9 contain protein isolated from beads bound with 2 µg of PCSK9 or ANGPTL3 antibody. Negative CTRL represents proteins from unbound beads (N = 3). Two non-adjacent parts of the same blot and same acquisition were merged as indicated by the double line. The uncropped blot is presented in the Supplementary Material. IP:ANGPTL3 and IP:PCSK9 sample in the cell medium are relative to proteins isolated from 2 mL of growth medium using beads bound with 2 µg of ANGPTL3 or PCSK9 antibody, respectively. Negative CTRL contains proteins isolated from a 2 mL medium using unbound beads. Anti-PCSK9 and anti-ANGPTL3 represent the antibodies hybridized on the membrane (N = 3). The green arrow indicates the ANGPTL3 band, and the red arrow indicates the PCSK9 band. Immunoglobulins (IgGs) are indicated with a blue bracket. (**B**) shows densitometries relative to the co-immunoprecipitations of cell lysates and cell media. (**C**) shows the immunofluorescence of HepG2 cells formalin-fixed in standard growth conditions. Cell nuclei are stained with DAPI and shown in blue. ANGPTL3 is shown in green. PCSK9 is shown in red. The merged red-green image shows co-localization as yellow-orange spots. Regions positive for red/green colocalization are displayed as white spots. Images were acquired at 63× magnification. The scale bar is 50 µm as a reference. MK = marker, KDa = kilodaltons, IP:NoAb = immunoprecipitation with no antibody, IP:ANGPTL3 = immunoprecipitation with ANGPTL3 antibody, IP:PCSK9 = immunoprecipitation with PCSK9 antibody.

Our investigation was prompted by the observation that circulating PCSK9 levels are reduced in patients with genetic loss of ANGPTL3 function [16], suggesting a potential functional interplay between these two key regulators of lipid metabolism. Given the reciprocal relationship observed between ANGPTL3 and PCSK9, we hypothesized that this association might involve the formation of a common protein complex. We therefore sought to determine whether ANGPTL3 and PCSK9 physically associate under physiological conditions; to demonstrate this hypothesis, we performed co-immunoprecipitation (Co-IP) experiments. We verified the ANGPTL3-PCSK9 association in both cell lysate and cell medium, using the human hepatic cell line HepG2 as a model. As shown in Figure 1A, in our initial pilot experiment, in the anti-ANGPTL3 immunoprecipitated sample (IP:ANGPTL3), we detected mature PCSK9 (mPCSK9). Similarly, in the anti-PCSK9 immunoprecipitated sample (IP:PCSK9), we detected ANGPTL3, although the band was fainter in the input sample. Interestingly, a comparable pattern was also observed in the cell medium, where the weaker signal was detected in the IP:ANGPTL3 sample rather than in the IP:PCSK9 sample. To obtain an initial indication of the stoichiometric relationship between the two proteins, we quantified the relative band intensity of ANGPTL3 in IP:PCSK9 and the intensity of the PCSK9 band in IP:ANGPTL3 in both cell lysate and cell medium. In both samples, we found that around 65% of ANGPTL3 immunoprecipitates with PCSK9 (mean 0.678 ± 0.09 for cell lysate; mean 0.634 ± 0.11 for cell medium). Conversely, around 30% of PCSK9 immunoprecipitates with ANGPTL3 (mean 0.298 ± 0.019 for cell lysate; mean 0.287 ± 0.06 for cell medium) (Figure 1B). 

These findings supported our initial hypothesis and prompted us to further investigate the potential association between ANGPTL3 and mature PCSK9 and to characterize the conditions that regulate their association. In parallel with the co-immunoprecipitation experiments, we performed immunofluorescence analyses in HepG2 cells to investigate the subcellular localization of ANGPTL3 and PCSK9 and assess their potential co-localization. As shown in Figure 1C, the signal corresponding to ANGPTL3 (green) is predominantly located in the perinuclear area and diffused in the cytoplasm with some secretion granules. The signal corresponding to PCSK9 (red) is widely spread in the cytoplasm and secretion granules. To further characterize this preliminary qualitative evaluation of merged immunofluorescence images, we performed a computational colocalization analysis using a custom Python-based approach to identify overlapping fluorescence signals. The analysis suggested a widespread intracellular overlap between ANGPTL3 and PCSK9 signals, mainly in the cytoplasm and secretory granules of HepG2 cells. Taken together, these findings support the existence of an ANGPTL3–PCSK9-containing complex that can be detected in both intracellular and extracellular compartments. 

Following experimental observations supporting a potential ANGPTL3-PCSK9 interaction, we next performed an in silico structural analysis using two independent predictive platforms to assess the formation and stability of the complex. We used AlphaFold2 and ZDOCK software to graphically model the ANGPTL3-PCSK9 complex. We used the sequences corresponding to the full-length and the mature forms of the two proteins as input for computational analysis. We analyzed different stoichiometric combinations: (1) 1:1 ANGPTL3-PCSK9, (2) 2:1 ANGPTL3-PCSK9, and (3) 3:1 ANGPTL3-PCSK9. As shown in Supplementary Figure S1A,B, the 1:1 ANGPTL3-PCSK9 complex shows too much uncertainty in the N-terminus ANGPTL3 domain; conversely, in the 3:1 stoichiometry, the third ANGPTL3 subunit would wind the N-terminus alpha-helix in reverse, resulting in loose contact with the other 2 subunits and PCSK9. On the contrary, when we modeled the interaction between full-length ANGPTL3 and mature PCSK9 in a 2:1 ratio (pTM = 0.55; ipTM = 0.50; pLDDT = 79), this complex showed the highest prediction ratios, considering recent conformational findings of ANGPTL3 as a circulating trimer (Supplementary Figure S1C). 

To support these results, we compared the score obtained in the 2:1 ANGPTL3-PCSK9 complex analyses with existing ANGPTL3-containing complexes: the ANGPTL3 trimer [24] and the 2:1 ANGPTL3-ANGPTL8 complex [25], and the ANGPTL3-ANGPTL8 complex found in a stoichiometry of 2:1 (Supplementary Figure S1D). Overall, the scores obtained for the ANGPTL3–PCSK9 complex were comparable to those reported for previously characterized ANGPTL3-containing complexes in the literature, supporting the reliability of our analysis and providing further confidence in the predicted 2:1 stoichiometry of the ANGPTL3–PCSK9 complex.

**Figure 2:**
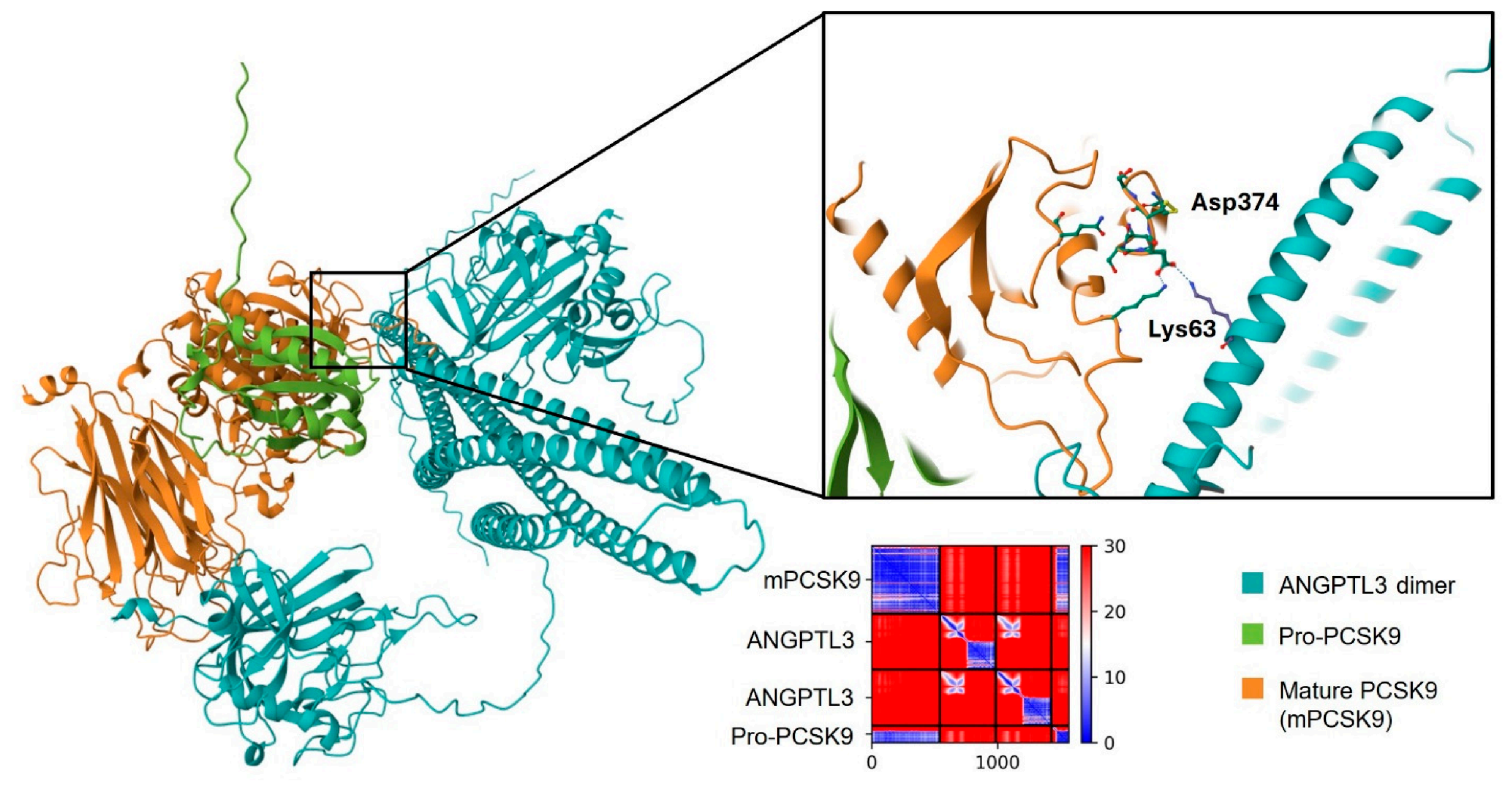
In silico prediction of ANGPTL3-PCSK9 interaction. AlphaFold2 modeled the ANGPTL3:PCSK9 complex in a 2:1 ratio. A circulating ANGPTL3 dimer with a similar structure was used as the template, together with mature PCSK9, in which the propeptide blocks the catalytic site., similar to the mature PCSK9, where pro-PCSK9 blocks its protease site. Mature PCSK9 interacts with both ANGPTL3 proteins at two specific sites, the N-terminus and C-terminus, respectively. The pro-PCSK9 occupies the center of the complex, interacting solely with mature PCSK9. In the zoomed-in model, PCSK9 p.Asp374 appears to be obstructed by p.Lys63 in one of the two ANGPTL3 molecules, potentially causing steric hindrance and reducing affinity for LDLR.

Moreover, the same interaction was independently predicted by ZDOCK [22] (Figure 2). In this analysis of the ANGPTL3-PCSK9 complex, the two ANGPTL3 chains are predicted to interact with PCSK9 at residues p.Glu211–Phe21 and p.Phe515-Glu516. These residues fall within the PCSK9 binding region for LDLR [26]. In particular, p.Asp374 in the PCSK9 chain is predicted to interact with p.Lys63 in the ANGPTL3 chain, as detailed in Figure 2. Overall, the in silico analyses provide convergent evidence supporting the potential formation of an ANGPTL3–PCSK9 complex, consistent with the experimental findings from Co-IP and immunofluorescence analyses.

### 3.2. Feeding and Fasting Conditions Influence the Detection of the ANGPTL3–PCSK9 Complex in HepG2 Cells and Human Plasma

**Figure 3:**
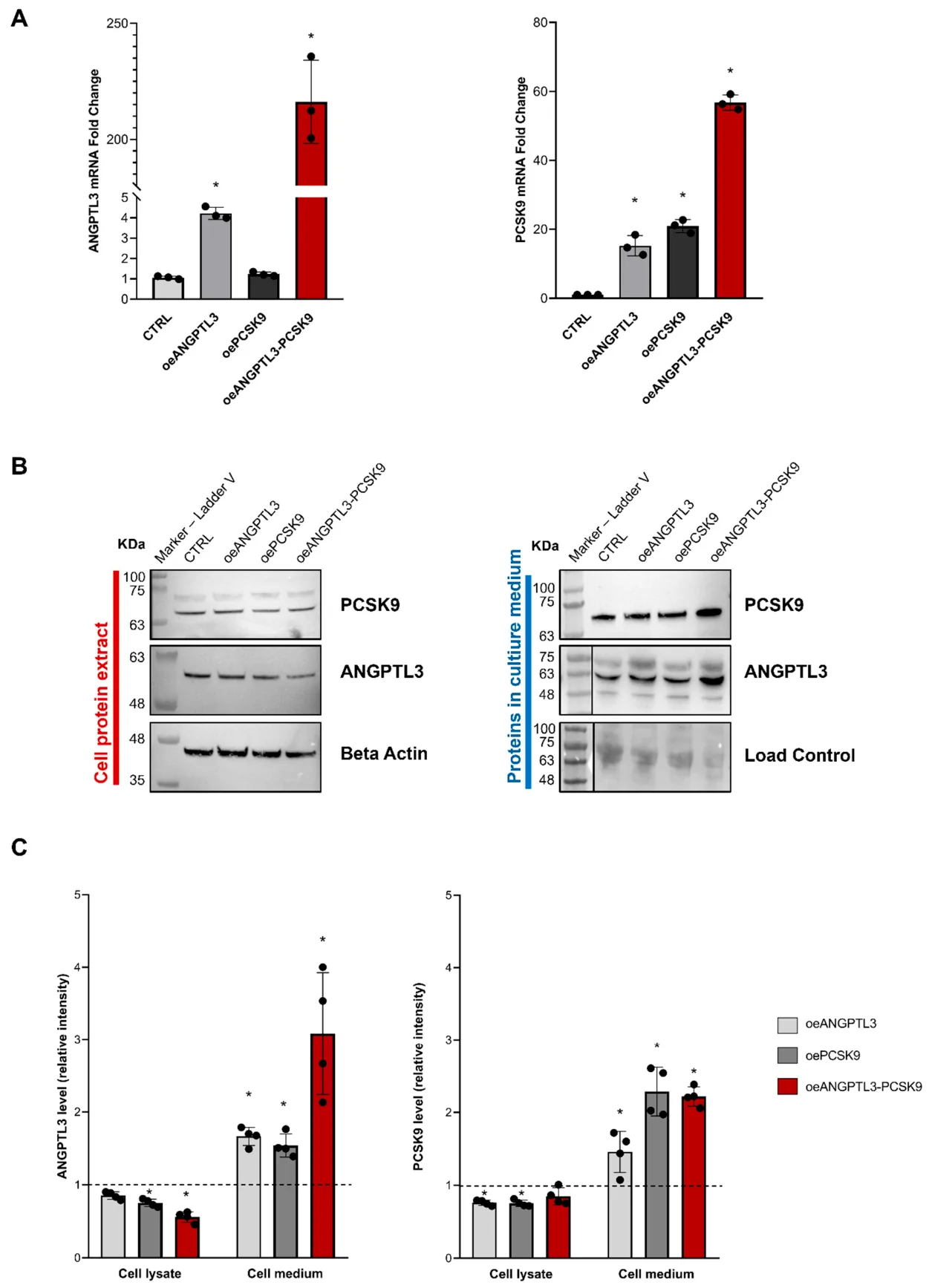
The overexpression of PCSK9 increases intracellular ANGPTL3 expression. (**A**) shows ANGPTL3 mRNA levels in HepG2 cells under the different experimental conditions; results are expressed in mRNA expression levels compared to the control condition. Asterisks indicate significant differences within the same overexpression condition (*p* < 0.05). Samples were compared with the CTRL group using Student’s *t*-test (N = 4). (**B**) shows Western blot membranes of ANGPTL3 and PCSK9 in 30 µg of total protein extract (intracellular proteins) or in 2 mL of culture medium (extracellular proteins). Two non-adjacent parts of the same blot and same acquisition were merged as indicated by the double line. The uncropped blot is presented in the Supplementary Material (N = 4). (**C**) shows the densitometries of ANGPTL3 and PCSK9 in intracellular or extracellular proteins compared to CTRL. Asterisks indicate a statistically significant difference (*p* < 0.05) in the Student *t*-test. MK = marker, KDa = kilodaltons.

Following previous results supporting the existence of an ANGPTL3-PCSK9 complex, we searched for potential mutual regulation between the two proteins. To this aim, we generated HepG2 cell lines overexpressing *ANGPTL3* (oeANGPTL3), overexpressing *PCSK9* (oePCSK9), or both genes (oeANGPTL3-PCSK9). The overexpression model was chosen in order to enhance the mechanisms of interaction involving one, the other, or both proteins. Consistently, all overexpressing cells showed higher levels of *ANGPTL3* or *PCSK9* mRNA than control cells. Surprisingly, the overexpression of *ANGPTL3* increased the mRNA levels of *PCSK9*, suggesting that ANGPTL3 may regulate PCSK9 at the transcriptional level. Conversely, PCSK9 overexpression does not affect ANGPTL3 mRNA levels. Moreover, the overexpression of both genes increases *ANGPTL3* expression more than 200-fold and *PCSK9* expression 60-fold compared with the control (Figure 3A). Regarding protein levels, oeANGPTL3 did not increase intracellular ANGPTL3 levels; conversely, in the cell medium, ANGPTL3 levels increased by about 70%. A similar pattern was observed in oePCSK9 cells; intracellular PCSK9 levels did not increase, whereas secreted PCSK9 increased ~2-fold. Surprisingly, the simultaneous overexpression of both genes has an additive effect on ANGPTL3 transcript and protein levels, with ANGPTL3 accumulating in the cell medium at 3-fold higher levels (Figure 3B,C). The additive increase in protein expression observed upon simultaneous overexpression of *ANGPTL3* and *PCSK9* is consistent with a coordinated regulation of the two proteins and, together with previous evidence showing reduced PCSK9 levels in the absence of ANGPTL3 [16], supports the possibility of a reciprocal relationship between ANGPTL3 and PCSK9.

Since ANGPTL3 and PCSK9 are known to be regulated by nutritional status, we investigated how nutrients affect the ANGPTL3-PCSK9 complex. To simulate fasting and feeding conditions, HepG2 cells were grown in nutrient-rich or nutrient-poor medium. The compositions of the two media are detailed in Supplementary Table S2. Specifically, to replicate feeding conditions, we cultured cells in nutrient-poor medium and, after 48 h, switched to nutrient-rich medium for 4 h; conversely, transitioning from nutrient-rich to nutrient-poor medium was used to mimic fasting (details of the experiment are reported in Supplementary Figure S2). We further validated that our simulated feeding and fasting conditions recapitulate physiological feeding and fasting by assessing ANGPTL8 secretion and the formation of the ANGPTL3–ANGPTL8 complex (Supplementary Figure S3A) [25]. ANGPTL8 was not detected in the cell medium under fasting conditions, whereas it was secreted under feeding conditions and co-immunoprecipitated with ANGPTL3, confirming that the system behaves as expected.

The levels of ANGPTL3 and PCSK9 proteins under all conditions, both in the media and intracellularly, were analyzed by Western blotting and reported in Supplementary Figure S4. Overall, ANGPTL3 varies widely between feeding and fasting conditions. Its intracellular and circulating levels were reduced in fasting and increased in feeding conditions. Conversely, PCSK9 did not consistently change under feeding conditions. It was predominantly secreted in fasting conditions. Surprisingly, PCSK9 overexpression increased both intracellular and extracellular ANGPTL3 levels, particularly under nutrient-rich conditions. As expected, oeANGPTL3-PCSK9 showed the highest ANGPTL3 levels, particularly in the cell medium, as previously observed under basal conditions.

To analyze the behavior of the ANGPTL3-PCSK9 complex under different nutritional conditions, we repeated the Co-IP for ANGPTL3 and PCSK9 in the culture medium of HepG2 cells grown in different nutritional conditions. 

**Figure 4:**
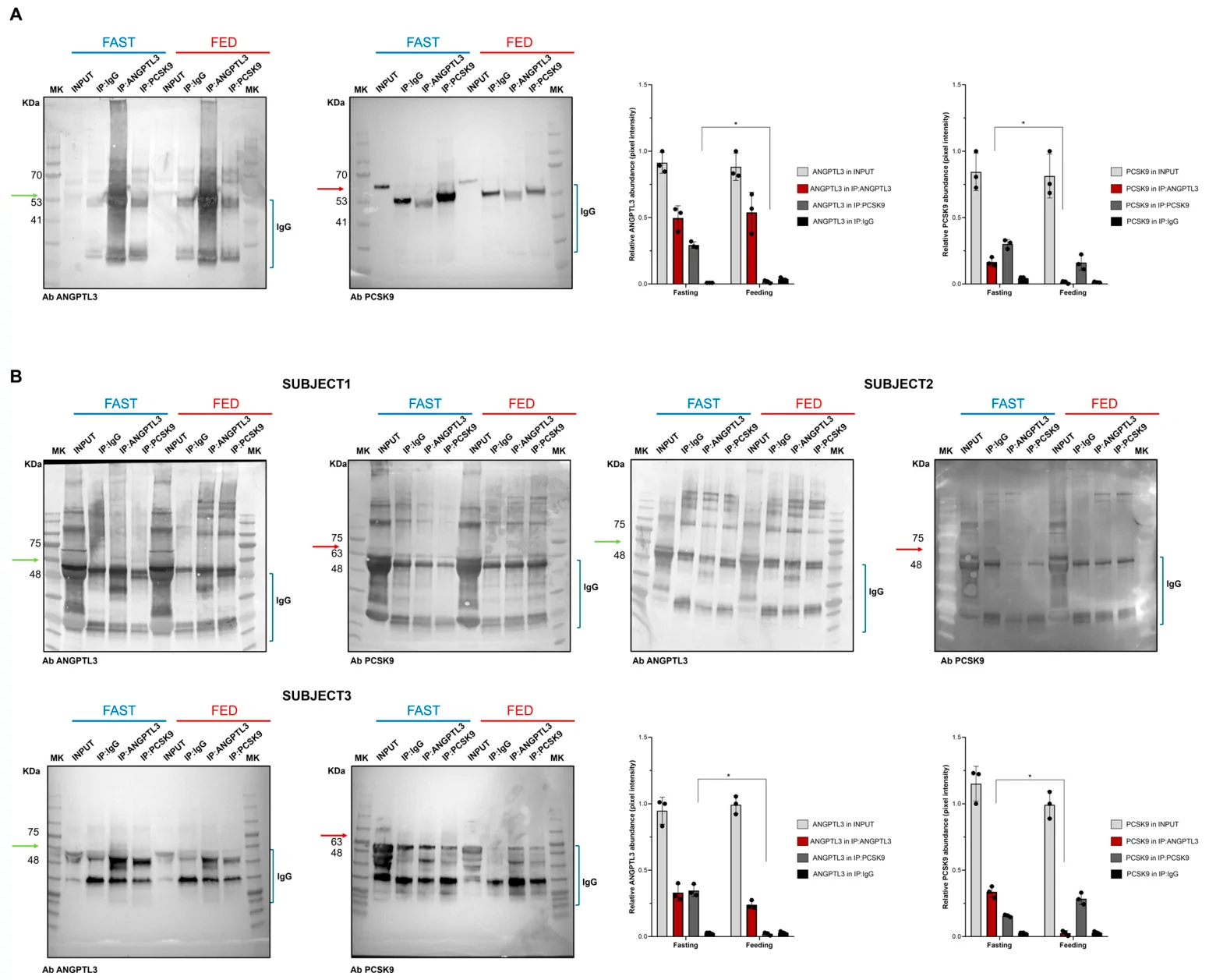
ANGPTL3 and PCSK9 co-immunoprecipitate preferentially under fasting conditions in HepG2 culture medium and human plasma. (**A**) shows the immunoprecipitation of ANGPTL3 and PCSK9 in fasting and feeding conditions (N = 3) and relative densitometries. Green arrow indicates ANGPTL3 band, red arrow indicates PCSK9 band. Immunoglobulins (IgGs) are indicated with a blue bracket. Asterisks indicate significant differences in the same overexpressing condition (*p* < 0.05). Sample was compared to the CTRL using Student’s *t*-test. Western blots in Panel A were run in gradient (4–12%) Bis-Tris MOPS buffer. (**B**) shows all the Western blots of Co-immunoprecipitations in the plasma of N = 3 subjects in conditions of fasting and feeding. The green arrow indicates ANGPTL3, the red arrow indicates PCSK9, and immunoglobulins (IgGs) are indicated by a blue bracket. Asterisks indicate significant differences in the same overexpressing condition (*p* < 0.05). Sample was compared to the CTRL using Student’s *t*-test. MK = marker, KDa = kilodaltons, IP:IgG = immunoprecipitation with anti-immunoglobulin antibody, IP:ANGPTL3 = immunoprecipitation with ANGPTL3 antibody, IP:PCSK9 = immunoprecipitation with PCSK9 antibody.

As shown in Figure 4A, reciprocal Co-IP in culture medium under fasting conditions was consistent with the presence of an ANGPTL3–PCSK9-containing complex. Surprisingly, in feeding conditions, the interaction was not detected by co-immunoprecipitation. We also quantified the relative intensity of the immunoprecipitated bands. In the IP:ANGPTL3 sample, around 50% of ANGPTL3 in INPUT is immunoprecipitated (0.496 ± 0.09), whereas 30% of PCSK9 in INPUT is immunoprecipitated in IP:ANGPTL3 only in fasting conditions (0.293 ± 0.02). The opposite is observed in the IP:PCSK9 sample, where around 30% of PCSK9 in INPUT is immunoprecipitated (0.284 ± 0.08), whereas around 15% of ANGPTL3 in INPUT is immunoprecipitated in IP:PCSK9, but only in fasting conditions (0.166 ± 0.03).

To assess whether the nutrient-dependent changes observed in HepG2 cells could also be detected in a physiological setting, we performed reciprocal Co-IP experiments in plasma samples from healthy donors collected under fasting and feeding conditions. Plasma samples were obtained before and after an oral fat load [6]. Specifically, we analyzed samples from three healthy donors (one male and two females) under fasting conditions and 4.5 h after a 980 kcal standard meal. The postprandial time point was selected based on the reported plasma chylomicron peak after feeding [6]. Blood chemistry and anthropometric data of enrolled subjects are reported in Supplementary Table S1. Reciprocal Co-IP experiments were performed in all subjects under both nutritional conditions. As shown in Figure 4B, immunoprecipitation of either ANGPTL3 or PCSK9 revealed the presence of an ANGPTL3–PCSK9-containing complex in fasting plasma samples, whereas the interaction was not detected in feeding conditions. In the IP:ANGPTL3 samples, approximately 30% of ANGPTL3 present in INPUT was immunoprecipitated (0.285 ± 0.07), together with around 30% of PCSK9 in INPUT, although only under fasting conditions (0.347 ± 0.18). Conversely, in the IP:PCSK9 samples, approximately 20% of PCSK9 in INPUT was immunoprecipitated (0.221 ± 0.07), while around 30% of ANGPTL3 in INPUT was co-immunoprecipitated only in fasting conditions (0.337 ± 0.04). Previous studies have shown that ANGPTL3 can also form a 2:1 complex with ANGPTL8 in feeding conditions [25]. To further assess the physiological consistency of our experimental model, we therefore evaluated the presence of ANGPTL8 in IP:ANGPTL3 samples from human plasma. As shown in Supplementary Figure S3B, ANGPTL8 was not detected in *fasting* IP:ANGPTL3 samples, whereas a corresponding band became detectable under feeding conditions. Overall, these findings further support the presence of an ANGPTL3–PCSK9-containing complex under fasting conditions, consistently detected in both HepG2-derived samples and human plasma, suggesting that the observed interaction may be physiologically modulated by nutrient availability.

### 3.3. LDL Incubation Is Associated with Reduced ANGPTL3–PCSK9 Co-Localization

Literature data have established that PCSK9 plays a key role in regulating *LDLR* expression at the cell membrane [27]. In addition, both ANGPTL3 and PCSK9 are known to regulate LDL metabolism through distinct but interconnected metabolic pathways [17,26]. Based on these observations, we hypothesized that the association between ANGPTL3 and PCSK9 could potentially influence the availability of PCSK9 regions involved in LDLR recognition.

To preliminarily explore this possibility, we investigated whether LDL exposure could affect the apparent ANGPTL3–PCSK9 association. To this aim, HepG2 overexpressing cells were analyzed before and after a 4 h treatment with 10 µg/mL LDL by immunofluorescence and colocalization analysis to evaluate potential changes in the intracellular distribution and overlap of ANGPTL3 and PCSK9 signals.

**Figure 5:**
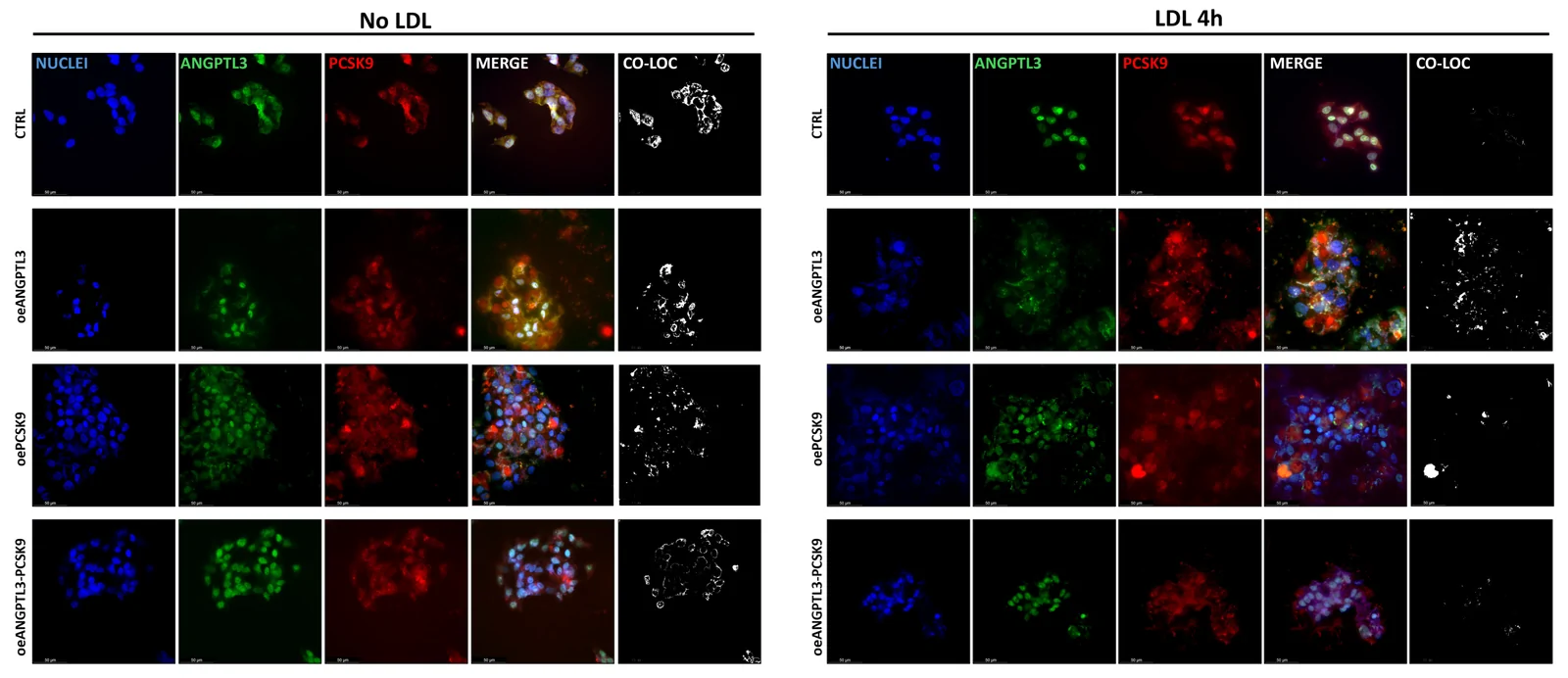
LDL treatment is associated with reduced ANGPTL3–PCSK9 co-localization. Immunofluorescence of HepG2 cells incubated with or without 10 µg/mL of LDL-C for 4 h. Cell nuclei are shown in blue, PCSK9 in red and ANGPTL3 in green. ANGPTL3-PCSK9 co-localization is represented in yellow in the merged figure. Regions positive for red/green colocalization are displayed as white spots.

As shown in Figure 5, CTRL cells displayed a marked intracellular co-localization between ANGPTL3 and PCSK9 under basal conditions, which was markedly reduced after 4 h of LDL incubation. In oeANGPTL3 cells, co-localization was still observed under basal growth conditions, although PCSK9 appeared predominantly distributed throughout the cytosol, whereas ANGPTL3 was mainly localized in the perinuclear area. Following 4 h of LDL treatment, the overlap between the two signals was no longer appreciable.

In oePCSK9 cells, co-localization appeared more limited under basal conditions and was mainly detectable within a subset of secretory vesicles. After LDL incubation, this overlap was markedly reduced. Similarly, in oeANGPTL3-PCSK9 cells, only limited co-localization was observed in secretory vesicles under basal conditions, whereas after 4 h of LDL exposure the two signals appeared largely separated.

Given the established role of PCSK9 in the regulation of LDLR membrane abundance, we evaluated LDLR levels at the cell membrane under basal growth conditions in CTRL, oeANGPTL3, oePCSK9 and oeANGPTL3-PCSK9 cells. As shown in Supplementary Figure S3C, no significant differences in membrane-associated LDLR abundance were observed among the different experimental conditions.

In parallel, we evaluated the presence of ANGPTL8 in conditioned medium following incubation with increasing LDL concentrations. As shown in Supplementary Figure S3D, ANGPTL8 became detectable after 4 h of LDL exposure at concentrations of 5 µg/mL and 10 µg/mL, whereas no detectable signal was observed at 20 µg/mL. Based on these findings, 10 µg/mL LDL was selected for the subsequent immunofluorescence colocalization experiments.

Overall, these observations indicate that LDL exposure is associated with a progressive reduction in ANGPTL3–PCSK9 co-localization across experimental conditions, suggesting that nutrient-related lipid stimuli may influence the intracellular association between the two proteins.

## 4. Discussion

In this work, we provide the first evidence consistent with the existence of an ANGPTL3–PCSK9-containing complex. This putative complex can be detected in HepG2 cells, culture medium, and human plasma, with an enrichment under fasting conditions.

These findings may help explain the observation by Fazio et al. [16] that FHBL2 patients show reduced levels of circulating PCSK9. If the overexpression of PCSK9 determines the increase in the intracellular levels of ANGPTL3, we might postulate that the absence of ANGPTL3 may contribute to reduced PCSK9 levels. Moreover, the existence of an ANGPLT3-PCSK9 complex may also offer a further interpretation of the hypocholesterolemic phenotype associated with ANGPTL3 deficiency [6]. Our findings raise the possibility that ANGPTL3–PCSK9 association may participate in nutrient-responsive regulation in hepatocytes. We also tried to quantitatively estimate the amount of protein immunoprecipitated in the ANGPTL3 and PCSK9 IP lanes, and we found that not all, but a fraction of ANGPTL3 and PCSK9 are in complexes and this fraction varies from 20 to 50%, with a wider variation in human plasma. This is consistent with metabolic regulation of the complex based on the metabolic status of the individual. In addition, the analysis of ratios in band intensity (e.g., (pixel intensity of ANGPTL3 in IP:PCSK9)/(pixel intensity of PCSK9 in IP:ANGPTL3)) has a value around 2, supporting a possible 2:1 stoichiometry of the complex. However, further biochemical studies are needed to definitely determine the complex stoichiometry.

It is well known that the PCSK9 protein represents a potent inhibitor of LDLR activity, as it reduces the availability of receptor protein on the cell surface [11,12]. As the structural prediction suggested, the ANGPTL3-PCSK9 complex might be inhibitory, especially for PCSK9. Most of the residues involved in the interaction of PCSK9 with ANGPTL3 are the crucial residues by which PCSK9 interacts with LDLR. These residues might be covered by the interaction with ANGPTL3, thus making PCSK9 unable to regulate LDLR; however, this remains hypothetical and was not functionally tested in the present study. Interestingly, when the ANGPTL3-PCSK9 complex is dissociated during feeding, ANGPTL3 is found associated with ANGPTL8. In this perspective, PCSK9 may integrate into the broader ANGPTL3–4–8 axis [4]. The ANGPTL3–4–8 system is a major regulator of triglyceride-derived fatty acid distribution between oxidative and storage tissues [4]. In fasting conditions, ANGPTL8 expression is low and ANGPTL3 has limited LPL-inhibitory activity, whereas after feeding ANGPTL8 is induced and forms a complex with ANGPTL3, enhancing inhibition of LPL in oxidative tissues and redirecting circulating triglyceride-derived fatty acids toward adipose tissue for storage [4]. Our observation that ANGPTL3–PCSK9 association is enriched during fasting and reduced after feeding raises the possibility that ANGPTL3 may dynamically redistribute between different protein partners according to nutrient availability. In this model, fasting would favor ANGPTL3 association with PCSK9, whereas feeding would shift ANGPTL3 toward the established ANGPTL3–ANGPTL8 axis. Although this interpretation remains hypothetical, it would place the ANGPTL3–PCSK9 association within an already established nutrient-responsive system controlling lipid partitioning. Moreover, the existence of a transient ANGPTL3-PCSK9 complex might be mechanistically interpreted as follows: a reduced accumulation of PCSK9 and ANGPTL3 in the culture medium may favor nutrient uptake by preserving maximal LDLR activity. Conversely, under feeding conditions, the reduced association and the increased secretion of both proteins may contribute to coordinated control of lipid fluxes. In particular, PCSK9 limits LDL uptake by modulating LDLR availability, whereas the ANGPTL3–ANGPTL8 complex inhibits LPL activity, thereby influencing the conversion of chylomicrons and VLDL into remnant particles. Overall, the interaction and reciprocal regulation between ANGPTL3 and PCSK9 may represent a finely tuned mechanism that adjusts hepatic nutrient uptake and export according to cellular and systemic demands. In this scenario, increases in PCSK9 expression may need to be counterbalanced by ANGPTL3, and co-overexpression of both proteins may engage a positive feedback on their expression, as observed in our experiments. Given this first evidence of a nutrient-dependent regulation of the novel ANGPTL3-PCSK9 complex and the leading regulatory role of the two proteins in the metabolism of lipoproteins, we decided to test the effect of low-density lipoprotein treatment on the complex. Surprisingly, LDL incubation alone was associated with loss of the co-localization pattern, a finding consistent with the possible dissociation of the complex. Under these conditions, we assessed whether ANGPTL8 was secreted into the culture medium. ANGPTL8 was detectable after 4 h at low and intermediate LDL concentrations, but not at high LDL concentration, possibly because high LDL triggers a more rapid switch to a fed-like state with earlier formation and degradation of the ANGPTL3–ANGPTL8 complex. As a further control, we also tested the expression of LDLR under basal conditions and when PCSK9 was overexpressed. LDLR levels did not change, probably due to the lack of ligand. In the absence of LDL particles, PCSK9 is not internalized and is unable to regulate LDL expression [11,12]. One possible interpretation is that reduced ANGPTL3–PCSK9 association after LDL incubation may increase PCSK9 availability for LDLR engagement, although this remains speculative. Dissociated ANGPTL3 may also form a complex with ANGPTL8, thus modulating the function of the lipoprotein lipase and regulating the influx of free fatty acids [3]. 

This nutrient-sensitive regulatory framework may also be relevant to metabolic conditions characterized by chronic nutrient excess, such as obesity and metabolic dysfunction-associated steatotic liver disease (MASLD). Obesity and insulin resistance are associated with impaired postprandial lipid handling, increased fatty acid flux, and altered hepatic lipoprotein metabolism [28]. Under these conditions, disruption of the normal fasting–feeding transition could theoretically modify the balance between ANGPTL3-containing complexes and the availability of PCSK9 for LDLR regulation. Such a mechanism could potentially influence both hepatic cholesterol uptake and peripheral triglyceride partitioning. However, the relationship between ANGPTL3 and hepatic lipid accumulation is complex. Importantly, human genetic ANGPTL3 deficiency is not associated with increased hepatic fat or a higher prevalence of hepatic steatosis, indicating that reduced ANGPTL3 activity per se does not necessarily promote hepatic lipid accumulation [7]. Conversely, pharmacological suppression of hepatic ANGPTL3 synthesis with vupanorsen was associated with increased liver fat, suggesting that the metabolic consequences may depend on the mechanism and cellular compartment through which ANGPTL3 is inhibited [29]. PCSK9 has also been linked to hepatic lipid homeostasis, although available evidence remains heterogeneous. Circulating PCSK9 has been associated with hepatic steatosis in human cohorts, whereas studies examining hepatic PCSK9 expression and PCSK9 inhibition in MASLD have yielded partly conflicting findings, suggesting that the relationship may depend on disease stage and metabolic context [30,31]. Unhealthy dietary patterns, particularly those characterized by excessive energy intake, ultra-processed foods, added sugars, and saturated fats, may contribute to the development and progression of MAFLD/MASLD. Conversely, dietary improvement, preferably within Mediterranean- or DASH-like patterns, together with a marked reduction or avoidance of alcohol intake, represents a cornerstone of non-pharmacological management [32].

The existence of a mutual regulation between ANGPTL3 and PCSK9 might have major effects on the management of the novel lipid-lowering therapies where ANGPTL3 and PCSK9 are core targets. As ANGPTL3 and PCSK9 are reciprocally regulated at the intracellular levels, the inhibition of one target could affect the other. For instance, while treatment with the monoclonal antibody against ANGPTL3, Evinacumab, determined a 49% reduction in LDL-C by acting directly on the circulating protein, the discontinued antisense oligonucleotide inhibiting ANGPTL3 at the intracellular level (Vupanorsen) reduces LDL-C to less than 10% [29,33]. Considering the interaction between ANGPTL3 and PCSK9, loss of intracellular ANGPTL3 may lead to an artifactual increase in PCSK9 activity, potentially compensating for the enhanced cholesterol clearance observed upon ANGPTL3 knockdown [29]. This effect may not occur when ANGPTL3 is inhibited with monoclonal antibodies, because the protein remains present, at least intracellularly, and may still modulate PCSK9 function. Alternatively, a fraction of PCSK9 may be sequestered in immune complexes containing ANGPTL3 and evinacumab. In this scenario, it is possible to speculate that the failure of vupanorsen to reduce LDL-C might be due to an ANGPTL3-free PCSK9 that could effectively inhibit LDLR recycling without a sufficient extracellular ANGPTL3 inhibition that accelerates LPL and EL activity and remnant metabolism. On the other hand, a double intracellular inhibition of ANGPTL3 and PCSK9 using RNAi technology could lead to effective LDL-C reduction, but potentially increased and damaging liver steatosis that could be determined by reduced VLDL secretion and increased LDL and remnants uptake. The potential ANGPTL3–PCSK9 association may also influence the pharmacological modulation of the PCSK9–LDLR axis. Monoclonal antibodies against PCSK9 act extracellularly by preventing circulating PCSK9 from binding to LDLR, whereas inclisiran reduces hepatic PCSK9 synthesis. If a fraction of PCSK9 is associated with ANGPTL3 under fasting conditions, nutrient-dependent changes in this association could theoretically modify the pool of PCSK9 available for LDLR engagement and, consequently, the amount of extracellular PCSK9 accessible to neutralizing antibodies. Conversely, intracellular PCSK9 silencing could affect both free and ANGPTL3-associated PCSK9 pools. However, the present study did not directly assess PCSK9–LDLR binding, LDLR recycling, or the response to PCSK9-directed therapies, and these implications remain hypothetical.

This study has several limitations and strengths. Although we obtained convergent evidence consistent with the hypothesis that ANGPTL3–PCSK9 can be present in the same protein complex, the present data do not establish direct binary binding between the two proteins. Co-immunoprecipitation demonstrates co-recovery within the same immunoprecipitable fraction but cannot distinguish direct interaction from indirect association mediated by additional proteins. Likewise, immunofluorescence supports partial spatial proximity but does not firmly prove a molecular interaction. Finally, the structural models are intended only as hypothesis-generating findings and do not provide experimental validation of stoichiometry or binding interfaces. Regarding the human data, although the limited number of human subjects was analyzed, we observed an-all-or-none effect; thus, we included three subjects as biological replicates. This data is also supported by the identification of an ANGPTL3-ANGPTL8 complex known in the literature [25], which is secreted only under feeding conditions and could represent a stimulus that favors complex dissociation. As a further limitation, we have not investigated in detail the intracellular pathways that regulate the formation and dissociation of the complex. Moreover, since the recent development of numerous drugs capable of inhibiting the expression of ANGPTL3 and PCSK9, most of the literature data concerning the intracellular role of ANGPTL3 and PCSK9 are based on knockdown or knockout models. Conversely, we decided to use an overexpression cellular model of the two proteins to better evaluate and identify the complex. Therefore, additional investigations are necessary to clarify the intracellular regulation and the wider metabolic implications of the observed ANGPTL3-PCSK9 complex.

## 5. Conclusions

In conclusion, this study, by employing three different experimental strategies, provides convergent evidence for a previously unrecognized ANGPTL3–PCSK9 association, detectable in HepG2 cells, culture medium, and human plasma, with enrichment under fasting conditions. Reciprocal regulation between ANGPTL3 and PCSK9 may be relevant to lipid-lowering medications targeting either pathway. Distinct approaches that neutralize circulating ANGPTL3 versus suppress its intracellular production could differentially affect PCSK9 abundance and activity, potentially contributing to heterogeneous LDL-C responses and suggesting that therapeutic sequencing or combination should be evaluated in light of intracellular cross-talk and sequestration dynamics. Future studies should define complex stoichiometry, dissect pathways controlling complex assembly/disassembly, and establish functional consequences under ligand-present conditions by quantifying PCSK9–LDLR engagement. Overall, the ANGPTL3–PCSK9 association emerges as a candidate regulatory node linking metabolic state to lipoprotein metabolism and warrants further mechanistic investigation.

## Data Availability

The original contributions presented in this study are included in the article/Supplementary Material. Further inquiries can be directed to the corresponding author.

## Supplementary Materials

The following supporting information can be downloaded at: https://www.mdpi.com/article/10.3390/biom16101531/s1: Supplementary Figure S1. In silico prediction of different ANGPTL3-PCSK9 interaction models. Supplementary Figure S2. *Feeding* and *Fasting* experiment setup. Supplementary Figure S3. ANGPTL8 binds ANGPTL3 in human plasma in *feeding* condition. Supplementary Figure S4. Nutrients modulate ANGPTL3 and PCSK9 levels-oePCSK9 increases ANGPTL3 expression. Supplementary Table S1. Anthropometric data of the three healthy subjects enrolled in the study for the Co-immunoprecipitation experiments. Supplementary Table S2. Medium composition for the nutrient-rich (DMEM) and nutrient-poor (F12) conditions. Supplementary Data S1: Protein Sequences for in silico interaction assay. The original Western blot images can be found in the Supplementary Materials.
