# Supplementary material for: A Nutrient-Dependent ANGPTL3–PCSK9-Containing Complex in HepG2 Cells and Human Plasma
Source: Biomolecules. 2026 Oct 20;16(10):1531. doi: 10.3390/biom16101531 (PMC13702755; doi:10.3390/biom16101531)
Supplement: Supplementary file 1 [file biomolecules-16-01531-s001.zip › biomolecules-4583093-supplementary.pdf]

SUPPLEMENTARY MATERIAL

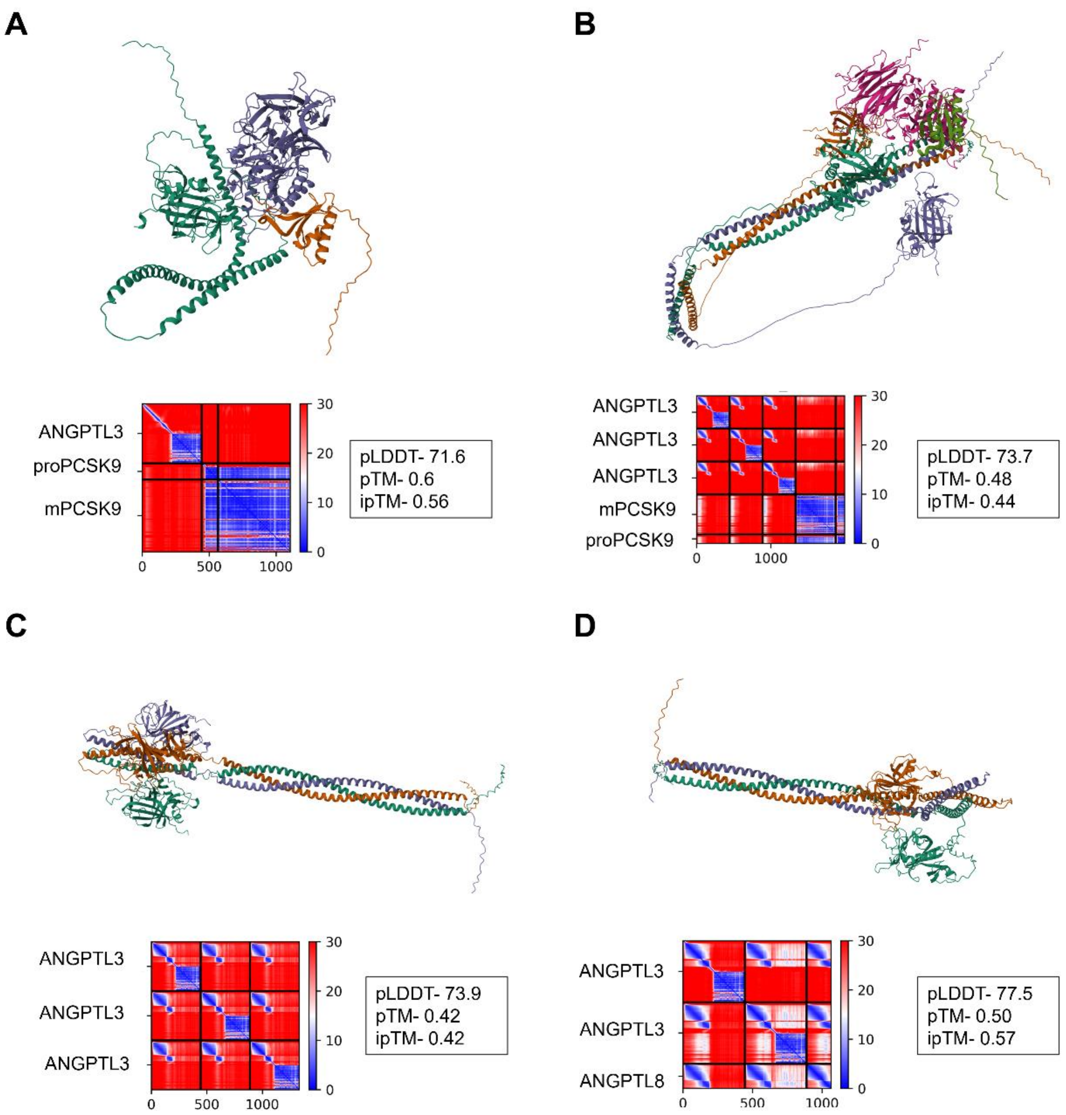

Supplementary Figure S1. In silico prediction of different ANGPTL3-PCSK9 interaction models -Panel A shows the potential interaction between ANGPTL3 full-length and mature PCSK9 (mPCSK9) with PCSK9 propetide (proPCSK9) . **Panel B** shows the interaction between ANGPTL3 and mature PCSK9 at a stoichiometry 3:1. **Panel C** shows the interaction of the demonstrated ANGPTL3 trimer. **Panel D** shows the interaction of the demonstrated ANGPTL3:ANGPTL8 complex at a stoichiometry of 2:1.

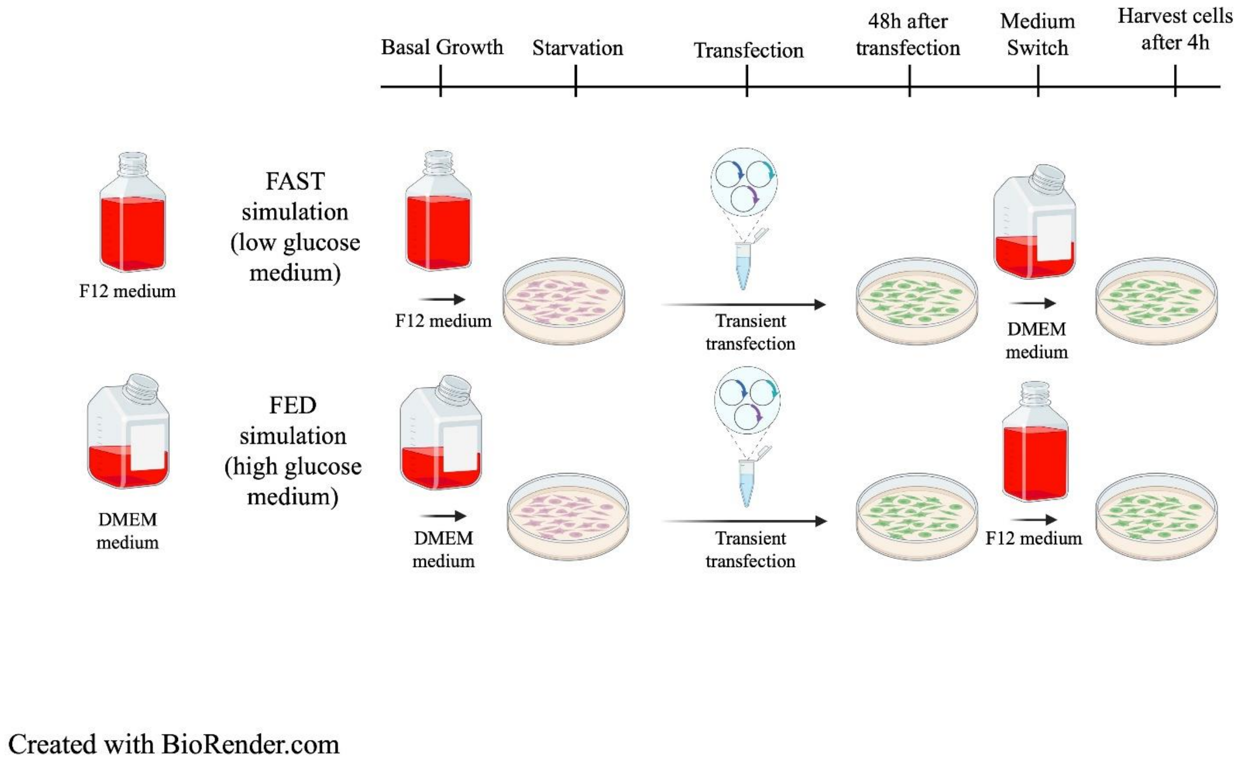

Supplementary Figure S2. ***Feeding*** **and** ***Fasting*** **experiment setup**. HepG2 cells at 70% confluence were starved in either DMEM or in F12 and transiently transfected with *ANGPTL3, PCSK9* or both genes for 48h. After 48 h, the medium was switched to DMEM in cells previously grown in F12 (simulating *feeding)* and to F12 in cells previously grown in DMEM (simulating *fasting*). Cells were kept for an additional 4h in fresh DMEM or F12.

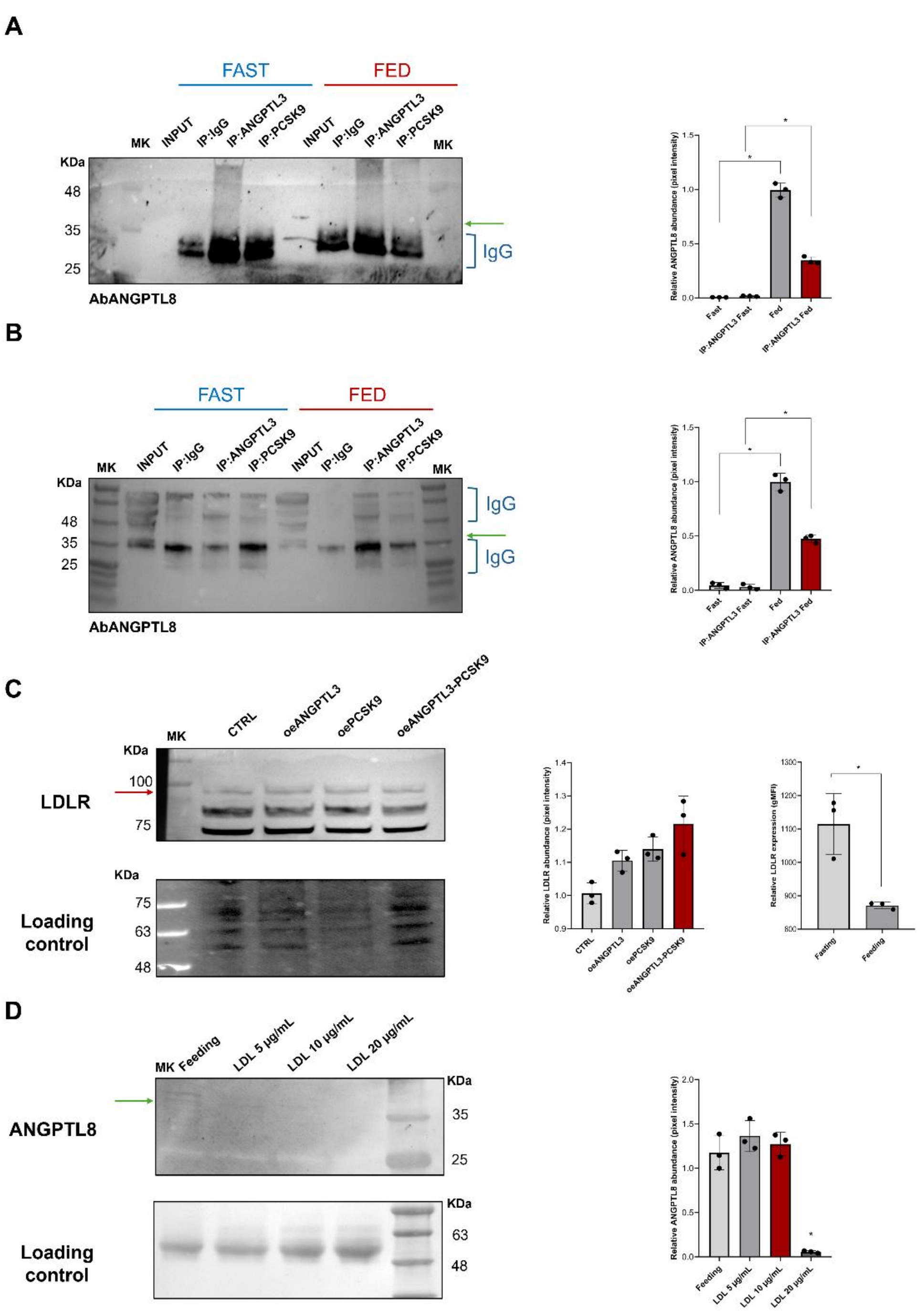

Supplementary Figure S3. ANGPTL8 binds ANGPTL3 in human plasma in *feeding* condition – Panel A shows ANGPTL8 in co-immunoprecipitation experiments performed on culture media under fasting and fed conditions (N = 3). The green arrow indicates the ANGPTL8 band. Immunoglobulins are indicated in blue brackets Asterisks indicates a statistically significant difference (P< 0.05) in the Student T-test. **Panel B** shows Plasma Co-immunoprecipitations of N=3 subjects in conditions of fasting and feeding. The figure shows the anti-ANGPTL8 antibody hybridization, the green arrow points at the ANGPTL8 band. Immunoglobulins are indicated in blue brackets, ANGPTL8 interacts with ANGPTL3 only in *feeding* conditions in human plasma.

Panel C shows LDLR expression in HepG2 cells in basal growth conditions in the CTRL, oeANGPTL3, oePCSK9 and oeANGPTL3-PCSK9 samples. (N=3). Statistically significant difference was set at P<0.05, densitometries were compared using the Student's t-test. LDLR expression under fasting and fed conditions was determined in CTRL cells using flow cytometry. Statistically significant difference was set at P<0.05, mean fluorescence among samples were compared using two-way ANOVA (N=3) **Panel D** shows ANGPTL8 secretion in the medium in HepG2 cells in the *feeding* condition or in HepG2 cells treated with increasing concentrations of LDLs. MK = marker, KDa = kilodaltons, IP:IgG = immunoprecipitation with anti-immunoglobulin antibody, IP:ANGPTL3 = immunoprecipitation with ANGPTL3 antibody, IP:PCSK9 = immunoprecipitation with PCSK9 antibody.

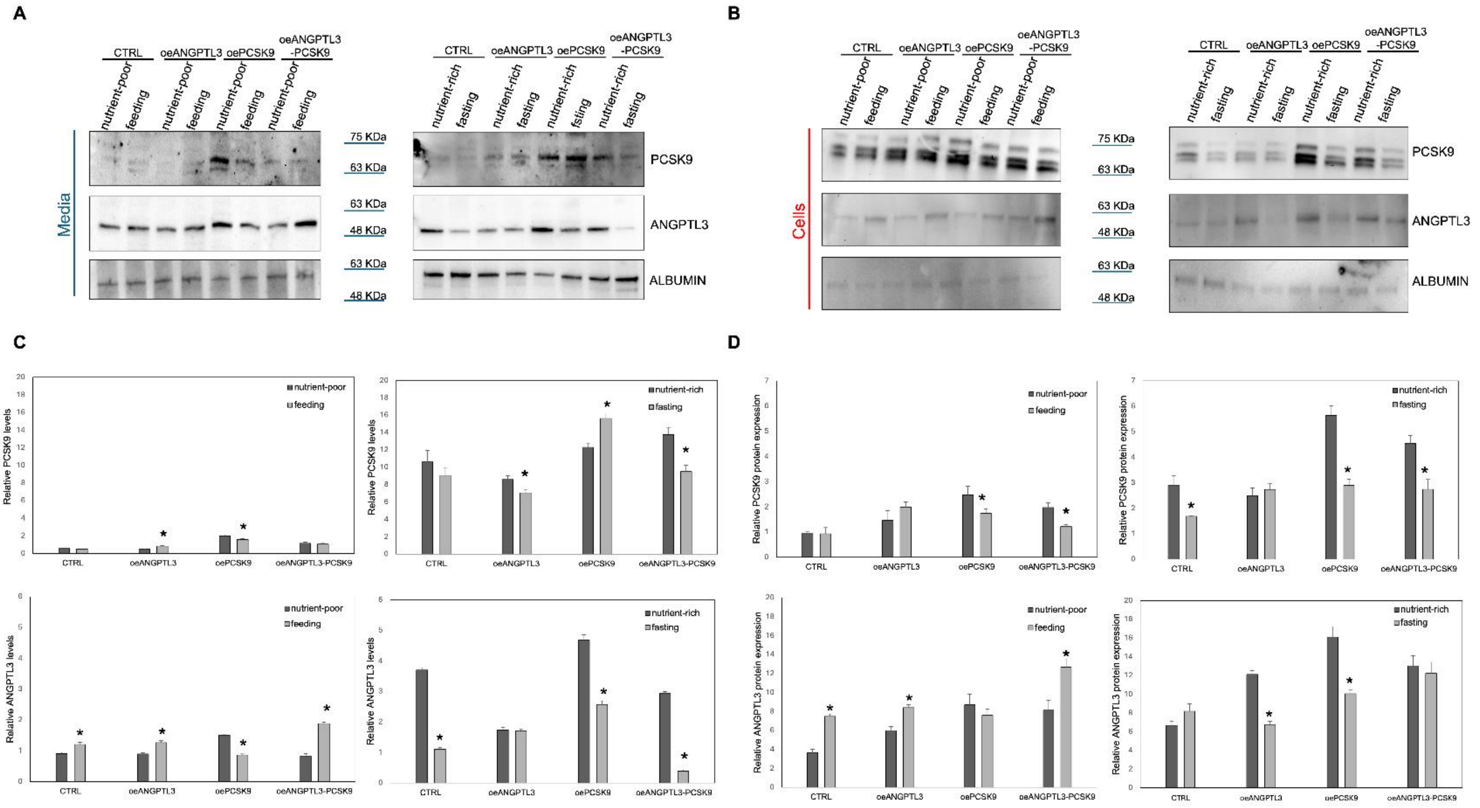

Supplementary Figure S4. Nutrients modulate ANGPTL3 and PCSK9 levels - oePCSK9 increases ANGPTL3 expression. Panel A shows a representative western blot of the culture medium. Proteins were extracted from 2 mL of FBS-free medium from cells cultured under nutrient-poor or nutrient-rich conditions, and simulating feeding condition (switching for 4h from nutrient-poor to nutrient-rich medium), or fasting condition (switching for 4h from nutrient-rich to nutrient-poor medium). **Panel B** shows the representative western blot analysis of 30 µg of total protein extract from cell lysate growth in the nutrient-poor or -rich medium and in *feeding* and *fasting* conditions. **Panels C** and **D** represent the densitometric analysis of three independent experiments (N=3). Data are shown as relative levels (for the media) and expression (for the cells) normalized for the quantity of the albumin. Asterisks indicate statistically significant change (p<0.05), test applied: Student's t-test.

	Subject 1	Subject 2	Subject 3
Age (y)	30	37	43
Sex (M/F)	M	F	F
Height (cm)	187	152	165
Weight (kg)	94	56	65
BMI	26,9	24,2	23,9
Waist circumference (cm)	96	88	82

Supplementary Table S1. Anthropometric data of the three healthy subjects enrolled in the study for the Co-immunoprecipitation experiments.

DMEM HIGH GLUCOSE		
**Components**	**Concentration (mg/L)**	**mM**
**Amino Acids**		
Glycine	30	0,4
L-Alanyl-Glutamine	862	3,9723501
L-Arginine hydrochloride	84	0,39810428
L-Cystine 2HCl	63	0,20127796
L-Histidine hydrochloride-H2O	42	0,2
L-Isoleucine	105	0,8015267
L-Leucine	105	0,8015267
L-Lysine hydrochloride	146	0,7978142
L-Methionine	30	0,20134228
L-Phenylalanine	66	0,4
L-Serine	42	0,4
L-Threonine	95	0,79831934
L-Tryptophan	16	0,078431375
L-Tyrosine disodium salt dihydrate	104	0,39846742
L-Valine	94	0,8034188
**Vitamins**		
Choline chloride	4	0,028571429
D-Calcium pantothenate	4	0,008385744
Folic Acid	4	0,009070295
Niacinamide	4	0,032786883
Pyridoxine hydrochloride	4	0,019417476
Riboflavin	0,4	0,00106383
Thiamine hydrochloride	4	0,011869436
i-Inositol	7,2	0,04
**Inorganic Salts**		
Calcium Chloride (CaCl2) (anhyd.)	200	1,8018018
Ferric Nitrate (Fe(NO3)3"9H2O)	0,1	2,48E-04
Magnesium Sulfate (MgSO4) (anhyd.)	97,67	0,8139166
Potassium Chloride (KCl)	400	5,3333335
Sodium Bicarbonate (NaHCO3)	3700	44,04762
Sodium Chloride (NaCl)	4750	81,89655
Sodium Phosphate monobasic (NaH2PO4-H2O)	125	0,9057971
**Other Components**		
D-Glucose (Dextrose)	4500	25
HEPES	5958	25,033613
Phenol Red	15	0,039851222

Ham F12 Kaighn Modification			
**Components**	**Concentration (mg/L)**	**mM**	**Fraction to DMEM**
**Amino Acids**			
Glycine	15	0,2	0,50
L-Alanine	18	0,20224719	-
L-Arginine hydrochloride	422	2	5,02
L-Asparagine-H2O	30	0,2	-
L-Aspartic acid	26,6	0,2	-
L-Cysteine hydrochloride-H2O	70	0,39772728	-
L-Glutamic Acid	29	0,19727892	-
L-Glutamine	29	2	-
L-Histidine hydrochloride-H2O	45,8	0,21809523	-
L-Isoleucine	7,88	0,060152672	-
L-Leucine	26,2	0,2	-
L-Lysine hydrochloride	73	0,3989071	0,50
L-Methionine	8,96	0,06013423	0,30
L-Phenylalanine	9,92	0,060121212	0,15
L-Proline	69	0,6	-
L-Serine	21	0,2	0,50
L-Threonine	23	0,19327731	0,24
L-Tryptophan	4,1	0,020098038	0,26
L-Tyrosine disodium salt dihydrate	13,5	0,051526718	0,13
L-Valine	23	0,1965812	0,24
**Vitamins**			
Biotin	0,07	2,87E-04	-
Choline chloride	14	0,1	3,50
D-Calcium pantothenate	0,5	0,001048218	0,13
Folic Acid	1,3	0,002947846	0,33
Niacinamide	0,037	3,03E-04	0,01
Pyridoxine hydrochloride	0,06	2,91E-04	0,02
Riboflavin	0,04	1,06E-04	0,10
Thiamine hydrochloride	0,3	8,90E-04	0,08
Vitamin B12	1,4	0,00103321	-
i-Inositol	18	0,1	2,50
**Inorganic Salts**			
Calcium Chloride (CaCl2) (anhyd.)	102	0,9189189	0,51
Cupric sulfate (CuSO4-5H2O)	0,002	8,00E-06	-
Ferric sulfate (FeSO4-7H2O)	0,8	0,002877698	-
Magnesium Chloride (anhydrous)	49,7	0,5231579	-
Magnesium Sulfate (MgSO4) (anhyd.)	192	1,6	1,97
Potassium Chloride (KCl)	285	3,8	0,71
Sodium Bicarbonate (NaHCO3)	2500	29,761906	0,68
Sodium Chloride (NaCl)	7530	129,82759	1,59
Sodium Phosphate dibasic (Na2HPO4) anhydrous	115,5	0,8133803	-
Sodium Phosphate monobasic (NaH2PO4) anhydrous	59	0,42446044	0,47
Zinc sulfate (ZnSO4-7H2O)	0,144	5,00E-04	-
**Other Components**			
D-Glucose (Dextrose)	1260	7	0,28
Hypoxanthine Na	4	0,030534351	-
Lipoic Acid	0,21	0,001019417	-
Phenol Red	3	0,007970245	-
Putrescine 2HCl	0,32	0,001987578	-
Sodium Pyruvate	220	2	-
Thymidine	0,7	0,002892562	-

Supplementary Table S2. Medium composition for the nutrient-rich (DMEM) and nutrient-poor (F12) conditions. Concentrations are expressed in mg/dL and nM. Fold change of media composition is calculated in comparison with DMEM.

Supplementary Data S1: Protein Sequences for in silico interaction assay

ANGPTL3

>sp|Q9Y5C1|17-460

SRIDQDNSSFDSLSPEPKSRFAMLDDVKILANGLLQLGHGLKDFVHKTKGQINDIFQKLN

IFDQSFYDLSLQTSEIKEEEKELRRTTYKLQVKNEEVKNMSLELNSKLESLLEEKILLQQ

KVKYLEEQLTNLIQNQPETPEHPEVTSLKTFVEKQDNSIKDLLQTVEDQYKQLNQQHSQI

KEIENQLRRTSIQEPTEISLSSKPRAPRTTPFLQLNEIRNVKHDGIPAECTTIYNRGEHT

SGMYAIRPSNSQVFHVYCDVISGSPWTLIQHRIDGSQNFNETWENYKYGFGRLDGEFWLG

LEKIYSIVKQSNYVLRIELEDWKDNKHYIEYSFYLGNHETNYTLHLVAITGNVPNAIPEN

KDLVFSTWDHKAKGHFNCPEGYSGGWWWHDECGENNLNGKYNKPRAKSKPERRRGLSWKS

QNGRLYSIKSTKMLIHPTDSESFE

PCSK9

ProPCSK9

>sp|Q8NBP7|31-152

QEDEDGDYEELVLALRSEEDGLAEAPEHGTTATFHRCAKDPWRLPGTYVVVLKEETHLSQ

SERTARRLQAQAARRGYLTKILHVFHGLLPGFLVKMSGDLLELALKLPHVDYIEEDSSVF

AQ

Mature PCSK9

>sp|Q8NBP7|153-692

SIPWNLERITPPRYRADEYQPPDGGSLVEVYLLDTSIQSDHREIEGRVMVTDFENVPEED

GTRFHRQASKCDSHGTHLAGVVSGRDAGVAKGASMRSLRVLNCQGKGTVSGTLIGLEFIR

KSQLVQPVGPLVVLLPLAGGYSRVLNAACQRLARAGVVLVTAAGNFRDDACLYSPASAPE

VITVGATNAQDQPVTLGTLGTNFGRCVDLFAPGEDIIGASSDCSTCFVSQSGTSQAAAHV

AGIAAMMLSAEPELTLAELRQRLIHFSAKDVINEAWFPEDQRVLTPNLVAALPPSTHGAG

WQLFCRTVWSAHSGPTRMATAVARCAPDEELLSCSSFSRSGKRRGERMEAQGGKLVCRAH

NAFGGEGVYAIARCCLLPQANCSVHTAPPAEASMGTRVHCHQQGHVLTGCSSHWEVEDLG

THKPPVLRPRGQPNQCVGHREASIHASCCHAPGLECKVKEHGIPAPQEQVTVACEEGWTL

TGCSALPGTSHVLGAYAVDNTCVVRSRDVSTTGSTSEGAVTAVAICCRSRHLAQASQELQ

ANGPTL8

>sp|Q6UXH0|22-198

APMGGPELAQHEELTLLFHGTLQLGQALNGVYRTTEGRLTKARNSLGLYGRTIELLGQEV

SRGRDAAQELRASLLETQMEEDILQLQAEATAEVLGEVAQAQKVLRDSVQRLEVQLRSAW

LGPAYREFEVLKAHADKQSHILWALTGHVQRQRREMVAQQHRLRQIQERLHTAALPA

Pixel-wise immunofluorescence merging and red/green colocalization analysis

Channel assignment: ch00 = blue, ch01 = green, ch02 = red.

```
# ============================================================
# Supplementary Code
# Pixel-wise immunofluorescence channel merging and
# red/green colocalization analysis with exclusion of
# blue-positive nuclear areas
# ============================================================
#
# This script performs RGB merging and pixel-wise colocalization
# analysis on three immunofluorescence channels.
#
# Expected channel naming:
#   ch00 = blue channel
#   ch01 = green channel
#   ch02 = red channel
#
# Main steps:
#   1. Load three TIFF images corresponding to blue, green, and red channels.
#   2. Extract the appropriate signal from grayscale or RGB TIFF files.
#   3. Perform background correction using a rolling-ball algorithm.
#   4. Convert each channel to 8-bit using percentile-based intensity scaling.
#   5. Apply moderate contrast enhancement and sharpening while preserving
#      a black background.
#   6. Perform pixel-wise red/green colocalization analysis.
```

```python
#   7. Exclude blue-positive pixels from the red/green colocalization mask.
#   8. Generate merged RGB images, colocalization masks, and control overlays.
#
# Required packages:
#   tifffile
#   scikit-image
#   numpy
#   matplotlib
#
# ============================================================

import os
import glob
import numpy as np
import tifffile as tiff
import matplotlib.pyplot as plt

from skimage.filters import threshold_otsu, unsharp_mask
from skimage import exposure
from skimage.restoration import rolling_ball


# ============================================================
# 1. USER-DEFINED PARAMETERS
# ============================================================

# Folder containing the three TIFF images
input_folder = "./"

# Common filename pattern used to identify the image set.
# The script will search for files containing this prefix and ch00/ch01/ch02.
# Example:
# target_prefix = "sample_condition_field_ch"
target_prefix = "sample_prefix_ch"

# Output folder
output_folder = "./output_colocalization_analysis"
os.makedirs(output_folder, exist_ok=True)

# Output filename prefix
output_prefix = "sample_colocalization_analysis"

# Channel assignment
blue_channel_id = "ch00"
green_channel_id = "ch01"
red_channel_id = "ch02"

# Background correction
rolling_ball_radius = 50
```

```python
# Percentile-based 8-bit conversion

low_percentile = 0.5

# Slightly more conservative scaling can be used for the red channel

# to avoid over-amplification of weak background or spillover signal.

red_high_percentile = 99.9

green_high_percentile = 99.8

blue_high_percentile = 99.8

# Contrast and sharpening parameters

clahe_clip = 0.010

sharp_radius = 1.0

sharp_amount = 0.60

gamma_value = 1.0

# More conservative red-channel enhancement

red_clahe_clip = 0.006

red_sharp_amount = 0.35

# Background preservation threshold

# Pixels below this value after 8-bit conversion are reset to zero.

black_threshold = 8

# Colocalization thresholds

# Otsu thresholds are calculated independently for each channel.

# Multiplicative factors allow adjustment of detection stringency.

threshold_factor_red = 0.85

threshold_factor_green = 0.80

threshold_factor_blue = 1.00


# ============================================================

# 2. FILE SELECTION

# ============================================================

all_files = glob.glob(os.path.join(input_folder, "**", "*"), recursive=True)

tif_files = [

    f for f in all_files

    if f.lower().endswith((".tif", ".tiff"))

]

target_files = [

    f for f in tif_files

    if target_prefix.lower() in os.path.basename(f).lower()

]

blue_files = [

    f for f in target_files

    if blue_channel_id.lower() in os.path.basename(f).lower()
```

```python
]

green_files = [
    f for f in target_files
    if green_channel_id.lower() in os.path.basename(f).lower()
]

red_files = [
    f for f in target_files
    if red_channel_id.lower() in os.path.basename(f).lower()
]

assert len(blue_files) > 0, "No blue channel file found."
assert len(green_files) > 0, "No green channel file found."
assert len(red_files) > 0, "No red channel file found."

blue_file = blue_files[0]
green_file = green_files[0]
red_file = red_files[0]

print("Selected files:")
print("Blue channel :", os.path.basename(blue_file))
print("Green channel:", os.path.basename(green_file))
print("Red channel  :", os.path.basename(red_file))


# ============================================================
# 3. IMAGE LOADING
# ============================================================

blue_img = tiff.imread(blue_file)
green_img = tiff.imread(green_file)
red_img = tiff.imread(red_file)

print("\nImage shapes and data types:")
print("Blue :", blue_img.shape, blue_img.dtype)
print("Green:", green_img.shape, green_img.dtype)
print("Red  :", red_img.shape, red_img.dtype)


# ============================================================
# 4. SIGNAL EXTRACTION
# ============================================================

def extract_signal(img, expected_channel):
    """
    Extract the signal from a grayscale or RGB image.

    Parameters
    ----------
    img : ndarray
```

```
        Input image.
    expected_channel : int
        Channel index to extract if the image is RGB:
        0 = red, 1 = green, 2 = blue.

    Returns
    -------
    ndarray
        Single-channel image.
    """
    if img.ndim == 2:
        return img

    if img.ndim == 3 and img.shape[-1] >= 3:
        return img[..., expected_channel]

    raise ValueError(f"Unsupported image format: {img.shape}")


B_raw = extract_signal(blue_img, expected_channel=2).astype(np.float32)
G_raw = extract_signal(green_img, expected_channel=1).astype(np.float32)
R_raw = extract_signal(red_img, expected_channel=0).astype(np.float32)

assert R_raw.shape == G_raw.shape == B_raw.shape, (
    "Input channels must have the same spatial dimensions."
)


# ============================================================
# 5. BACKGROUND CORRECTION
# ============================================================

def correct_background(img, radius=50):
    """
    Correct uneven background using rolling-ball subtraction.

    Parameters
    ----------
    img : ndarray
        Input single-channel image.
    radius : int
        Radius of the rolling-ball filter.

    Returns
    -------
    ndarray
        Background-corrected image.
    """
    background = rolling_ball(img, radius=radius)
    corrected = img - background
```

```python
    corrected[corrected < 0] = 0
    return corrected

R_corr = correct_background(R_raw, radius=rolling_ball_radius)
G_corr = correct_background(G_raw, radius=rolling_ball_radius)
B_corr = correct_background(B_raw, radius=rolling_ball_radius)

# ============================================================
# 6. 8-BIT INTENSITY SCALING
# ============================================================
def to_uint8_percentile(img, low_p=0.5, high_p=99.8):
    """
    Convert an image to 8-bit using percentile-based scaling.

    Parameters
    ----------
    img : ndarray
        Input image.
    low_p : float
        Lower percentile for scaling.
    high_p : float
        Upper percentile for scaling.

    Returns
    -------
    ndarray
        8-bit image.
    """
    img = img.astype(np.float32)

    high = np.percentile(img, high_p)

    if high <= 0:
        return np.zeros_like(img, dtype=np.uint8)

    low = np.percentile(img[img > 0], low_p) if np.any(img > 0) else 0

    if high <= low:
        low = 0

    x = (img - low) / (high - low + 1e-8)
    x = np.clip(x, 0, 1)

    return (x * 255).astype(np.uint8)

R8 = to_uint8_percentile(R_corr, low_percentile, red_high_percentile)
G8 = to_uint8_percentile(G_corr, low_percentile, green_high_percentile)
B8 = to_uint8_percentile(B_corr, low_percentile, blue_high_percentile)
```

```
merged_RGB_raw = np.dstack([R8, G8, B8]).astype(np.uint8)


# ============================================================
# 7. CONTRAST ENHANCEMENT WITH BLACK BACKGROUND PRESERVATION
# ============================================================

def enhance_channel_keep_black(
    ch8,
    clahe_clip=0.010,
    sharp_radius=1.0,
    sharp_amount=0.60,
    gamma_value=1.0,
    black_threshold=8
):
    """
    Enhance contrast and sharpness while preserving black background.

    Processing steps:
    - define a signal mask based on the black threshold;
    - apply CLAHE;
    - apply unsharp masking;
    - apply gamma correction;
    - reset background pixels to zero.

    Parameters
    ----------
    ch8 : ndarray
        8-bit input channel.
    clahe_clip : float
        CLAHE clip limit.
    sharp_radius : float
        Radius for unsharp masking.
    sharp_amount : float
        Amount for unsharp masking.
    gamma_value : float
        Gamma correction value.
    black_threshold : int
        Pixels below this value are treated as background.

    Returns
    -------
    ndarray
        Enhanced 8-bit channel.
    """
    x = ch8.astype(np.float32) / 255.0

    signal_mask = ch8 > black_threshold
```

```
    x = exposure.equalize_adapthist(
        x,
        clip_limit=clahe_clip
    )

    x = unsharp_mask(
        x,
        radius=sharp_radius,
        amount=sharp_amount,
        preserve_range=True
    )

    x = np.clip(x, 0, 1)
    x = x ** gamma_value

    x8 = (x * 255).astype(np.uint8)

    x8[~signal_mask] = 0
    x8[x8 < black_threshold] = 0

    return x8


R_enh = enhance_channel_keep_black(
    R8,
    clahe_clip=red_clahe_clip,
    sharp_radius=sharp_radius,
    sharp_amount=red_sharp_amount,
    gamma_value=gamma_value,
    black_threshold=black_threshold
)

G_enh = enhance_channel_keep_black(
    G8,
    clahe_clip=clahe_clip,
    sharp_radius=sharp_radius,
    sharp_amount=sharp_amount,
    gamma_value=gamma_value,
    black_threshold=black_threshold
)

B_enh = enhance_channel_keep_black(
    B8,
    clahe_clip=clahe_clip,
    sharp_radius=sharp_radius,
    sharp_amount=sharp_amount,
    gamma_value=gamma_value,
    black_threshold=black_threshold
)
```

```
merged_RGB_enhanced = np.dstack([R_enh, G_enh, B_enh]).astype(np.uint8)


# ============================================================
# 8. PIXEL-WISE COLOCALIZATION ANALYSIS
# ============================================================

def otsu_on_nonzero_pixels(ch):
    """
    Calculate Otsu threshold using only non-zero pixels.

    Parameters
    ----------
    ch : ndarray
        Input 8-bit channel.

    Returns
    -------
    float
        Otsu threshold. Returns zero if no signal is present.
    """
    if np.any(ch > 0):
        return threshold_otsu(ch[ch > 0])
    return 0


red_threshold_otsu = otsu_on_nonzero_pixels(R_enh)
green_threshold_otsu = otsu_on_nonzero_pixels(G_enh)
blue_threshold_otsu = otsu_on_nonzero_pixels(B_enh)

red_threshold = red_threshold_otsu * threshold_factor_red
green_threshold = green_threshold_otsu * threshold_factor_green
blue_threshold = blue_threshold_otsu * threshold_factor_blue

red_positive = R_enh > red_threshold
green_positive = G_enh > green_threshold
blue_positive = B_enh > blue_threshold

# Pixel-wise red/green overlap
coloc_RG = red_positive & green_positive

# Exclusion of blue-positive areas
coloc_RG_excluding_blue = coloc_RG & (~blue_positive)

print("\nThresholds:")
print("Red Otsu threshold   :", red_threshold_otsu)
print("Green Otsu threshold :", green_threshold_otsu)
print("Blue Otsu threshold  :", blue_threshold_otsu)

print("\nFinal thresholds:")
print("Red final threshold  :", red_threshold)
```

```
print("Green final threshold:", green_threshold)
print("Blue final threshold :", blue_threshold)

print("\nPixel-wise analysis:")
print("Red-positive pixels                      :", int(red_positive.sum()))
print("Green-positive pixels                    :", int(green_positive.sum()))
print("Blue-positive pixels                     :", int(blue_positive.sum()))
print("Red/green colocalized pixels             :", int(coloc_RG.sum()))
print("Red/green colocalized pixels excluding blue:", int(coloc_RG_excluding_blue.sum()))

print("\nPercentages relative to total image area:")
print("Total red/green colocalization             :", round(coloc_RG.mean() * 100, 3), "%")
print("Red/green colocalization excluding blue    :", round(coloc_RG_excluding_blue.mean() * 100, 3), "%")


# ============================================================
# 9. OUTPUT IMAGE GENERATION
# ============================================================

# RGB merge with red/green colocalization shown in white
merged_RGB_white_coloc_excluding_blue = merged_RGB_enhanced.copy()
merged_RGB_white_coloc_excluding_blue[coloc_RG_excluding_blue] = [255, 255, 255]

# Red/green colocalization mask
mask_RG = np.zeros_like(merged_RGB_enhanced)
mask_RG[coloc_RG] = [255, 255, 255]

# Red/green colocalization mask excluding blue-positive areas
mask_RG_excluding_blue = np.zeros_like(merged_RGB_enhanced)
mask_RG_excluding_blue[coloc_RG_excluding_blue] = [255, 255, 255]

# Blue-positive mask
blue_positive_mask = np.zeros_like(merged_RGB_enhanced)
blue_positive_mask[blue_positive] = [0, 0, 255]

# Control overlay
control_overlay = merged_RGB_enhanced.copy()
control_overlay[blue_positive] = [0, 0, 255]
control_overlay[coloc_RG_excluding_blue] = [255, 255, 255]


# ============================================================
# 10. SAVE TIFF OUTPUTS
# ============================================================

tiff.imwrite(
    os.path.join(output_folder, f"{output_prefix}_merged_RGB_raw.tif"),
    merged_RGB_raw,
    photometric="rgb"
)
```

```
tiff.imwrite(
    os.path.join(output_folder, f"{output_prefix}_merged_RGB_enhanced.tif"),
    merged_RGB_enhanced,
    photometric="rgb"
)

tiff.imwrite(
    os.path.join(output_folder, f"{output_prefix}_merged_RGB_white_coloc_excluding_blue.tif"),
    merged_RGB_white_coloc_excluding_blue,
    photometric="rgb"
)

tiff.imwrite(
    os.path.join(output_folder, f"{output_prefix}_mask_RG_colocalization.tif"),
    mask_RG,
    photometric="rgb"
)

tiff.imwrite(
    os.path.join(output_folder, f"{output_prefix}_mask_RG_colocalization_excluding_blue.tif"),
    mask_RG_excluding_blue,
    photometric="rgb"
)

tiff.imwrite(
    os.path.join(output_folder, f"{output_prefix}_blue_positive_mask.tif"),
    blue_positive_mask,
    photometric="rgb"
)

tiff.imwrite(
    os.path.join(output_folder, f"{output_prefix}_control_overlay.tif"),
    control_overlay,
    photometric="rgb"
)


# ============================================================
# 11. SAVE PNG PREVIEWS
# ============================================================

plt.imsave(
    os.path.join(output_folder, f"{output_prefix}_merged_RGB_raw_preview.png"),
    merged_RGB_raw
)

plt.imsave(
    os.path.join(output_folder, f"{output_prefix}_merged_RGB_enhanced_preview.png"),
    merged_RGB_enhanced
```

```
)

plt.imsave(
    os.path.join(output_folder, f"{output_prefix}_merged_RGB_white_coloc_excluding_blue_preview.png"),
    merged_RGB_white_coloc_excluding_blue
)

plt.imsave(
    os.path.join(output_folder, f"{output_prefix}_mask_RG_colocalization_excluding_blue_preview.png"),
    mask_RG_excluding_blue
)

plt.imsave(
    os.path.join(output_folder, f"{output_prefix}_blue_positive_mask_preview.png"),
    blue_positive_mask
)

plt.imsave(
    os.path.join(output_folder, f"{output_prefix}_control_overlay_preview.png"),
    control_overlay
)


# ============================================================
# 12. DISPLAY PREVIEWS
# ============================================================

plt.figure(figsize=(8, 8))
plt.imshow(merged_RGB_raw)
plt.title("Merged RGB raw")
plt.axis("off")
plt.show()

plt.figure(figsize=(8, 8))
plt.imshow(merged_RGB_enhanced)
plt.title("Merged RGB enhanced")
plt.axis("off")
plt.show()

plt.figure(figsize=(8, 8))
plt.imshow(merged_RGB_white_coloc_excluding_blue)
plt.title("Red/green colocalization excluding blue-positive areas")
plt.axis("off")
plt.show()

plt.figure(figsize=(8, 8))
plt.imshow(mask_RG_excluding_blue)
plt.title("Mask: red/green colocalization excluding blue")
plt.axis("off")
plt.show()
```

```
plt.figure(figsize=(8, 8))

plt.imshow(control_overlay)

plt.title("Control overlay")

plt.axis("off")

plt.show()
```
